# Chiral Binaphthalene Building Blocks for Self-Assembled Nanoscale CPL Emitters

**DOI:** 10.3390/molecules28083382

**Published:** 2023-04-11

**Authors:** Yu-Yu Hsieh, Jing-Jong Shyue, Yu-Chiang Chao, Ken-Tsung Wong, Dario M. Bassani

**Affiliations:** 1 Univ. Bordeaux, CNRS, Bordeaux INP, ISM UMR 5255, F-33400 Talence, France; 2 Department of Chemistry, National Taiwan University, Taipei 10617, Taiwan; 3 Research Center for Applied Sciences, Academia Sinica, Taipei 11529, Taiwan; 4 Department of Physics, National Taiwan Normal University, Taipei 11677, Taiwan; 5 Institute of Atomic and Molecular Science, Academia Sinica, Taipei 10617, Taiwan

**Keywords:** luminescence, circularly polarized luminescence, vesicles, binaphthalene, chirality

## Abstract

The introduction of biuret hydrogen-bonding sites onto chiral binaphthalene-based chromophores was investigated as a route to sub-micron-sized, vesicle-like aggregates endowed with chiroptical properties. The synthesis was conducted from the corresponding chiral 4,4′-dibromo-1,1′-bis(2-naphthol) via Suzuki–Miyaura coupling to afford luminescent chromophores whose emission spectrum could be tuned from blue to yellow-green through extension of the conjugation. For all compounds, the spontaneous formation of hollow spheres with a diameter of ca. 200–800 nm was evidenced by scanning electron microscopy, along with strong asymmetry in the circularly polarized absorption spectra. For some compounds, the emission also displayed circular polarization with values of *g*_lum_ = ca. 10^–3^ which could be increased upon aggregation.

## 1. Introduction

Hydrogen-bonding (H-B) interactions are widely used in natural and artificial systems to construct small, well-defined molecular assemblies in which the number and directionality of the complementary H-B donor and acceptor motifs are satisfied [1,2]. When this is not the case, either involuntarily or by design, pendant H-B units can lead to the formation of extended or infinite structures such as tubules, fibers, or sheets whose size usually extends beyond the micron scale [3,4,5]. Understandably, the self-assembly of well-defined H-B architectures whose size goes beyond that of a few molecules but remains well below the micron scale is more difficult as it requires a delicate balance between intermolecular forces pushing for the structure’s growth and those that confine it.

While investigating the self-assembly of rigid, π-conjugated chromophores appended with bis-urea (biuret) H-B units, we discovered that they spontaneously self-assemble into hollow spheres in organic solvents [6]. The spheres are characterized by a diameter comprised between 200 nm and 1 µm, and a thin wall of ca. 15–30 nm. Diffraction studies in solution revealed that the spheres are present in solution even in the absence of traces of water, and that they display Brownian motion rather than a propensity to aggregate [7]. The spontaneous self-assembly of well-defined spheres from small molecules is unusual and generally limited to phospholipids and polymersome classes of compounds. Instead, the bis-biuret motif is a surprisingly robust structural encoder for the programmed formation of vesicle-like hollow spheres that can accommodate a variety of photoactive cores, including porphyrins [8] and azobenzenes [9]. Furthermore, the nanoscale dimensions of the aggregates are particularly well adapted to the design of OLED materials in which each colored pixel is defined by a single aggregate, thereby opening the way to ultra-high-resolution displays in which color bleeding was minimized [10].

Progress beyond the control of the spatial distribution of the emissive material in OLEDs rests on the incorporation of additional functionalities such as error correction [11,12], improving the efficiency through thermally activated delayed fluorescence (TADF) [13,14], or the control of chirality for the emission of circularly polarized light (CPL) [15]. Interestingly, the biuret H-B motif smoothly accommodates TADF-active chromophores and a substantial improvement of the external quantum efficiency of the electroluminescent device can be obtained [16]. However, the incorporation of chiral elements has, to date, not been tested. The growing interest in CPL materials [17,18] for applications in 3-D displays [19], information processing [20,21], and anti-counterfeit inks [22] prompted us to explore whether the formation of hollow spheres from bis-biuret-appended chiral luminophores is possible. To this end, we selected the 1,1′-bisbinaphthyl chiral auxiliary to introduce axial chirality in rigid π-conjugated chromophores. Starting from asymmetric binaphthalene fluorophores with axial chirality, biuret motifs are introduced to induce the formation of self-assembled microstructures which may in turn affect the chiroptical properties of the chromophores.

**Figure 1:**
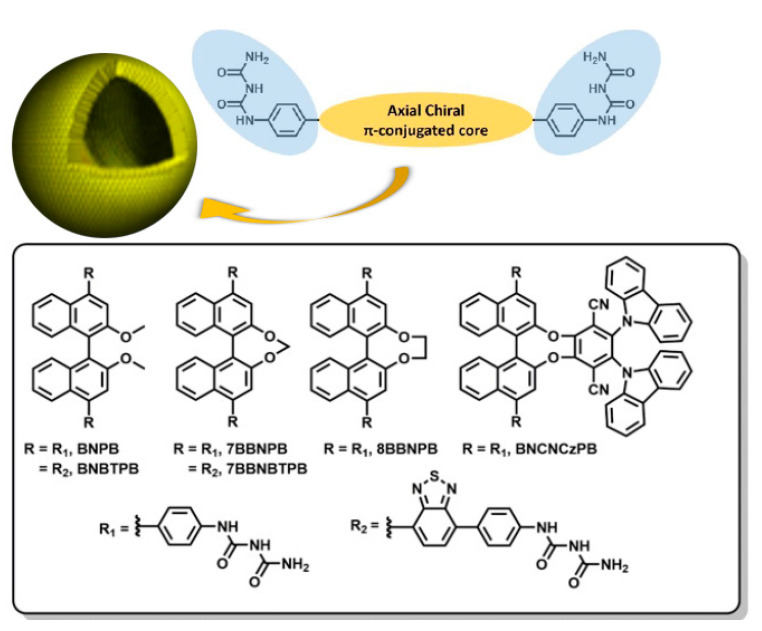
Structural design paradigm for the programmed spontaneous self-assembly of vesicle-like hollow spheres encoded through the introduction of phenyl-biuret motifs onto rigid π-conjugated platforms (**top**) and chemical structures of the compounds incorporating a chiral chromophore investigated herein (**bottom**).

The chemical structures of six target molecules are shown in Figure 1. To investigate systematically the effect of the open and bridged forms of the central bis-naphthalene chromophore on the chiroptical properties, we designed **BNPB**, **7BBNPB**, and **8BBNPB** for comparison. In addition, for **BNBTPB** and **7BBNBTPB**, the π-conjugation was extended by introducing benzothiadiazole moieties with the aim to tune the emission color from deep-blue to green-yellow. Previously, Pieters et al. investigated binaphthalene-based green CPL-TADF emitters using the chiral perturbation strategy and showed that they possess high photoluminescence quantum yields of 74% in toluene solution and moderate *g*_lum_ of 1.3 × 10^–3^ in CPL [23]. Therefore, we modified the chemical structure to obtain **BNCNCzPB** with the aim to demonstrate combined CPL and TADF characteristics in self-assembled vesicles.

## 2. Results

### 2.1. Synthesis

The preparation of the chiral binaphthol (BNOH) was reported previously. Briefly, bromination of 1-naphthylamine gave intermediate **2** in high yield (Scheme S1). Next, the aryl amine **2** was converted to the diazonium salt **3** through a Sandmeyer reaction. Reduction of compound **3** by sodium hydride to remove nitrogen afforded monomer **4** [24]. The latter led to the preparation of racemic mixtures of **BNOH** using copper-catalyzed oxidative coupling. To obtain enantiopure compounds, (1S)-(+)-10-camphorsulfonyl chloride was used as a chiral resolving agent, and the diastereomeric pairs were separated using silica chromatography. After hydrolysis using sodium hydroxide, the chiral intermediates (R)-**BNOH** and (S)-**BNOH** were obtained [25].

**Scheme 1:**
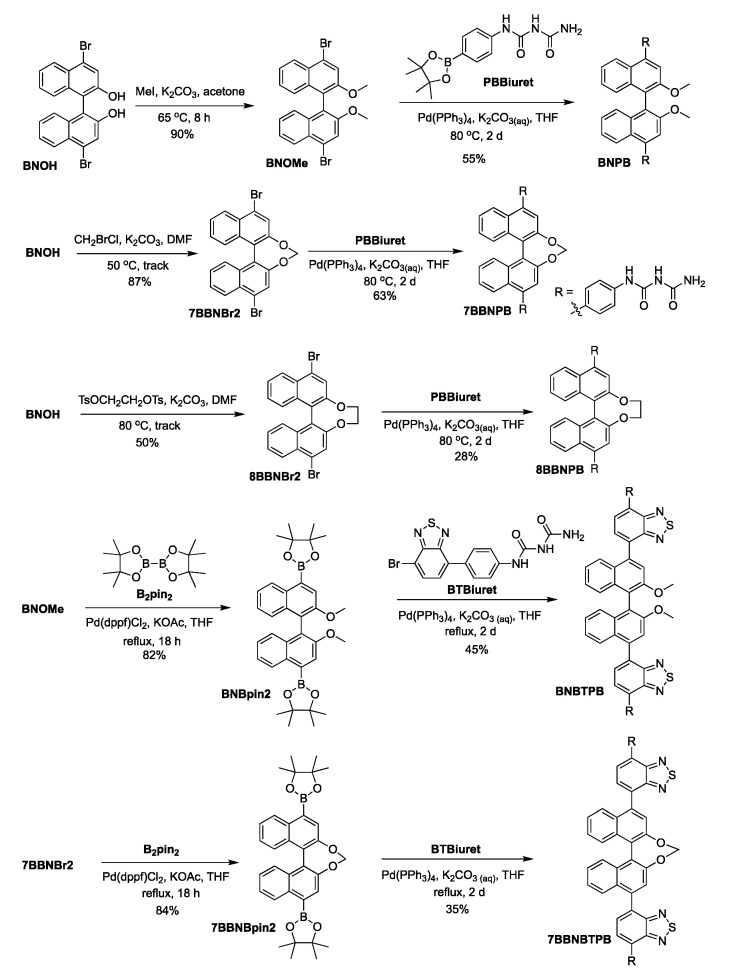
Synthetic methodology for the preparation of chiral bis-biuret chromophores.

The preparation of the chiral bis-biuret chromophores is presented in Scheme 1. Compound **BNOH** was converted to **BNOMe** using MeI, followed by Pd-catalyzed Suzuki–Miyaura coupling with **PBBiuret** to afford target molecule **BNPB**. For the bridged forms [26,27], the bisdiol was linked via an S_N_2 reaction using methylene or ethylene groups to lock the axial chiral conformation, followed by palladium-mediated Suzuki–Miyaura coupling with **PBBiuret** [28] to produce **7BBNPB** and **8BBNPB**. For the two yellow emitters, **BNOMe** or **7BBNBr2** were treated with bis(pinacolato)diboron in a palladium-catalyzed reaction to give the corresponding boronic ester derivatives. These were then reacted with **BTBiuret** through Suzuki–Miyaura coupling to obtain **BNBTPB** and **7BBNBTPB**.

**Scheme 2:**
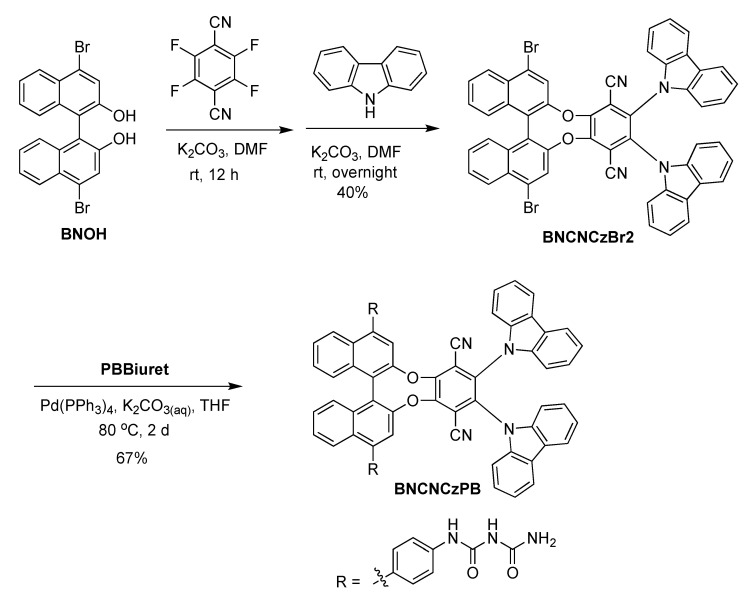
Synthetic methodology for the preparation of **BNCNCzPB** integrating two biuret units and a CPL-TADF chromophore.

For **BNCNCzPB**, the key intermediate **BNOH** underwent two S_N_Ar reactions with tetrafluoroterephthalonitrile and carbazole in a one-pot procedure to afford **BNCNCzBr2** [23]. Then, the target compound **BNCNCzPB** was obtained by Pd-mediated Suzuki–Miyaura coupling with **BNCNCzBr2** and **PBBiuret** (Scheme 2).

### 2.2. Characterization of Nanostructures

**Figure 2:**
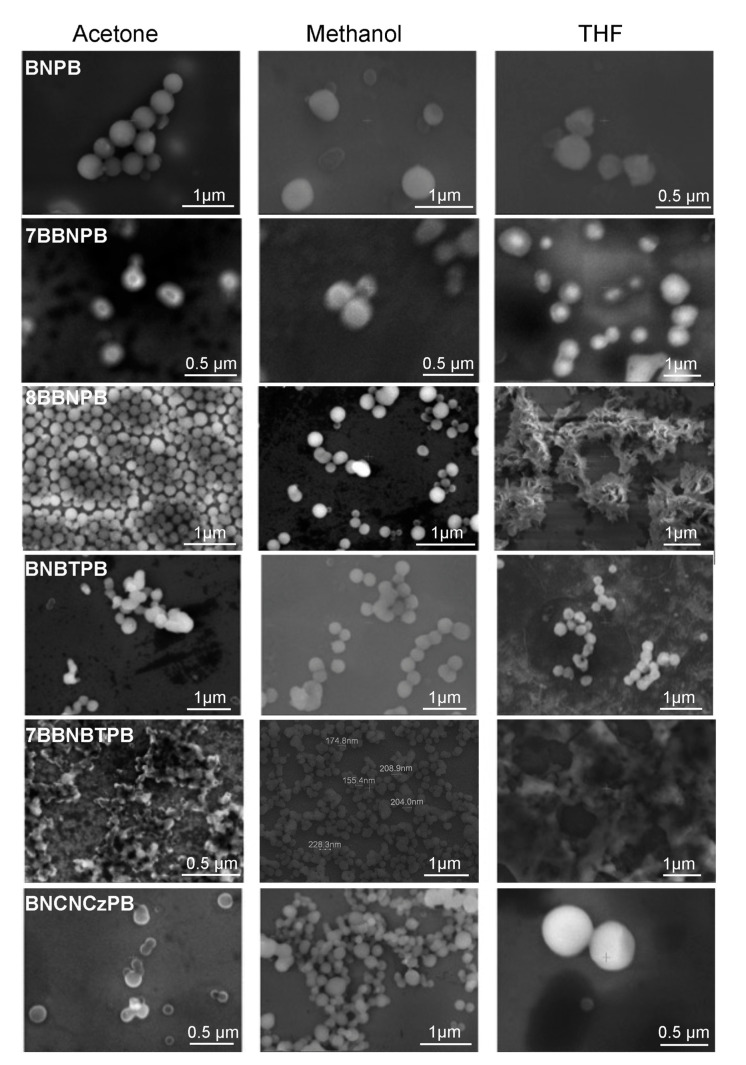
Scanning electron microscopy images of the aggregates observed upon deposition of dilute solutions (0.1 mM) of the BB and BN series of compounds onto glass substrates. The images were obtained in low-vacuum mode with a sample chamber pressure maintained at 0.45 Torr water using a differential pumping system.

To characterize the nanostructures formed by the BN series of molecules, the morphologies of the self-assembled aggregates were observed by SEM in low-vacuum mode with no conductive overcoat. The sample chamber pressure was maintained at 0.45 Torr water using a differential pumping system. The images of the structures deposited from different solvents onto SiO_2_ substrates are shown in Figure 2. For all six molecules, uniform and well-defined nanospheres in the size of 200–500 nm in acetone or methanol solutions were observed. However, when drop-cast from a THF environment, the compounds exhibit greater morphological variety and may organize into different nanostructures, such as vesicles (**BNPB**, **7BBNPB**, **BNBTPB**, and **BNCNCzPB**), films (**7BBNBTPB**), or clusters (**8BBNPB**).

### 2.3. Photophysical Properties

**Figure 3:**
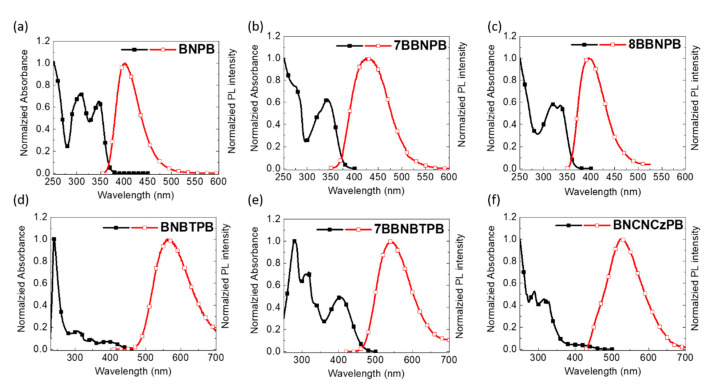
Electronic absorption and emission spectra of (**a**) **BNPB**, (**b**) **7BBNPB**, (**c**) **8BBNPB**, (**d**) **BNBTPB**, (**e**) **7BBNBTPB**, and (**f**) **BNCNCzPB** in THF solutions (10^–5^ M, λ_ex_ = 350 nm).

As shown in Figure 3 and summarized in Table 1, all the binaphthalene-based compounds possess a strong π-π* absorption band around 340 nm. Compared to the unlocked chromophore **BNPB**, the **7BBNPB** derivative in which the BiNAP unit is locked shows a bathochromic shift of the emission wavelength attributed to the greater planarity enforced by the methylene bridge along with through-bond conjugation [26,29,30,31]. Indeed, compound **8BBNPB**, possessing a longer and more flexible eight-member ring, has similar excited state behavior as **BNPB**.

As expected, the presence of an electron-accepting moiety (benzothiadiazole) extends the conjugation length of fluorophore. Thus, **BNBTPB** and **7BBNBTPB** have an absorption band near 400 nm and emission wavelengths near 530–550 nm vs. **BNPB** and **7BBNPB** which instead emit in the deep-blue.

All of the compounds are strongly emissive. Compared to the open-form **BNPB** (*F*_F_ = 0.81), the closed forms **7BBNPB** (*F*_F_ = 0.72) and **8BBNPB** (*F*_F_ = 0.66) have a more rigid structure, resulting in a decrease in the photoluminescence quantum yield. This is somewhat unexpected, as rigid molecules generally exhibit higher PLQYs compared to flexible molecules. In this case, we attribute this to the loss of planarization in the excited state due to the presence of the covalent linker. Similarly, the introduction of benzothiadiazole results (*F*_F_ = 0.28 and *F*_F_ = 0.42 for **BNBTPB** and **7BBNBTPB**, respectively) in a diminished photoluminescence efficiency [32,33].

**Table 1:** Summary of the photophysical properties of the BN and BB series of chromophores ^1^.

Compound	*λ*_abs_ (nm)	*ε* (10^4^ M^−1^cm^−1^)	*λ*_em_ (THF, nm)	*Φ*_THF_ ^2^ (%)
**BNPB**	307, 346	5.2, 4.1	402	81
**7BBNPB**	346	3.7	432	72
**8BBNPB**	335	5.3	396	66
**BNBTPB**	303, 344, 403	7.1, 3.3, 3.6	549	28
**7BBNBTPB**	318, 404	6.3, 4.1	538	42
**BNCNCzPB**	314, 328, 403	5.8, 5.1, 0.56	525	21 (28) ^3^

^1^ Measured in THF solution (10^–5^ M). ^2^ Fluorescence quantum yields in aerated THF solutions (10^−5^ M, *λ_e_*_x_ = 350 nm). Values in parentheses correspond to degassed solutions. ^3^ Measured using quinine sulfate in 0.105 M HClO_4_ solution as a secondary standard [34].

**Figure 4:**
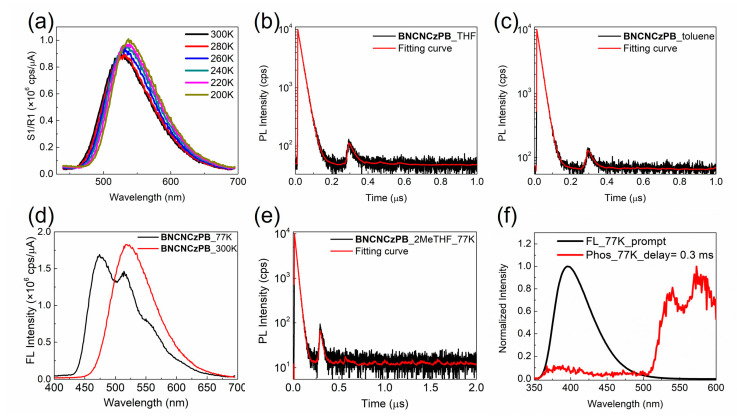
(**a**) Variable temperature emission spectra of **BNCNCzPB** in THF solution (10^–5^ M, λ_ex_ = 350 nm). Time-resolved decay of the emission from **BNCNCzPB** in degassed (**b**) THF or (**c**) toluene solution at 300 K (λ_ex_ = 370 nm, λ_em_ = 550 nm). (**d**) Emission spectra (λ_ex_ = 350 nm) and (**e**) time-resolved decay of the emission from **BNCNCzPB** in degassed 2-methyltetrahydrofuran solution (5 μM) at 77 K and 300 K (λ_ex_ = 370 nm, λ_em_ =550 nm). (**f**) Prompt (FL) and delayed (Phos) emission spectra of **BNPB** in frozen toluene solution (10^–5^ M, λ_ex_ =320 nm) at 77 K. The phosphorescence spectrum at 77 K is acquired after a 0.3 ms delay.

Compound **BNCNCzPB** exhibits an intense π-π* absorption band in the ultraviolet region, accompanied by a weaker intramolecular charge-transfer band around 400 nm (Figure 3f). The photoluminescence efficiency of this green-yellow emitter is only slightly increased from 21% to 28% in THF solutions after degassing, suggesting no or modest contribution from the triplet manifold through intersystem crossing (ISC). To verify the TADF character in **BNCNCzPB**, we employed variable-temperature photoluminescence spectra to observe the thermally activated ISC of the triplet excitons. Upon lowering the temperature, a decrease in the emission intensity is expected as the thermally activated ISC from the T_1_ to the S_1_ state is slowed. However, for **BNCNCzPB**, the PL intensity in degassed THF solution is enhanced upon decreasing the temperature (Figure 4a). Therefore, the TADF character is absent in this compound. In agreement with this, the time-resolved photoluminescence of **BNCNCzPB** in degassed THF or toluene solutions at room temperature only evidences a short-lived contribution with a lifetime of ca. 20 ns (Figure 4b,c). Furthermore, we dissolved **BNCNCzPB** in degassed 2-methyltetrahydrofuran solution (5 μM) to compare the emission at 77 K and 300 K. In contrast to the broad and structureless emission band observed at 300 K, the spectra at 77 K shows a hypsochromic shift from 525 to 475 nm along with vibronic fine structure (Figure 4d). This suggests that the intramolecular charge transfer state collapses to a localized emitting state as the temperature is lowered. No evidence of phosphorescence emission from the triplet state was observed (Figure 4e).

The situation is different for **BNPB**; upon lowering the temperature to 77 K, the broad PL emission centered at 387 nm is replaced by a structured emission at 540 nm (Figure 4f). The latter is assigned to phosphorescence emission from the triplet state. Thus, the introduction of phenyl-biuret motifs at 4- and 4′- of the binaphthalene chromophore results in a significant decrease in the energy of the triplet state. It is therefore likely that **BNCNCzPB**, with the same skeleton as **BNPB**, possesses a significantly lower triplet energy compared to the parent TADF chromophore **BNCNCz** which will be incapable of undergoing thermally activated ISC to populate the emissive S_1_ state.

### 2.4. Chiroptical Properties

**Figure 5:**
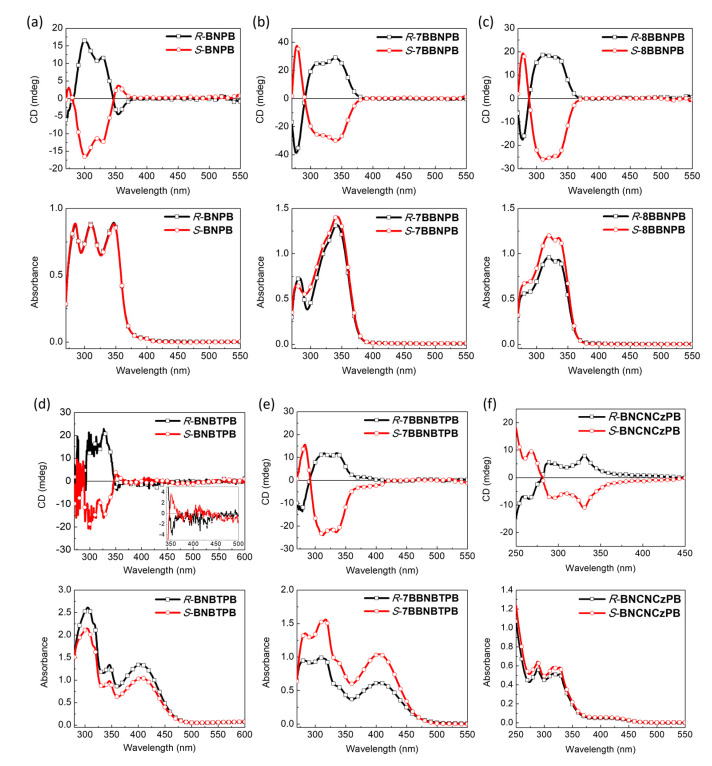
Electronic CD (**top**) and absorption (**bottom**) spectra of (**a**) *R*/*S*-**BNPB**, (**b**) *R*/*S*-**7BBNPB**, (**c**) *R/S*-**8BBNPB**, (**d**) *R/S*-**BNBTPB**, (**e**) *R/S-***7BBNBTPB**, and (**f**) *R/S*-**BNCNCzPB** (THF, 10^–5^ M). The inset in (**d**) is the magnified ECD spectra of *R/S*-**BNBTPB** between 350–500 nm.

To investigate the chiroptical properties of the BN series of compounds, we used CD and CPL spectra to analyze the relationship between the chirality in the ground and excited states and the electronic transitions to and from the excited state (Figure 5). The relatively large Stokes shift (Table 1) for both the BN and BB series suggests that the chromophores undergo relatively significant planarization during the relaxation of the Frank–Condon excited state. For all of the compounds, a near-perfect mirror–image relationship in the ECD spectra is observed for the enantiomers in terms of shape and peak maxima [35,36,37]. Nonetheless, we note that the absolute intensity is not identical for the BB series, which may be due to small differences in the formation of the aggregates upon dissolution of the material. The *R*-**BNPB** compound exhibits negative Cotton effects in the long-wavelength region, while other blue emitters (*R*-**7BBNPB** and *R*-**8BBNPB**) and green-yellow emitters *R*-**BNBTPB**, *R*-**7BBNBTPB**, and *R*-**BNCNCzPB** exhibit positive Cotton effects in the long-wavelength region. As the number of methylene units bridging the two naphthyl groups increases, the ECD intensity first increases and then decreases. This can be rationalized by considering the restricted torsional motion of the C–C bond joining the two naphthyl groups which is expected to be smallest for the single methylene spacer and larger in the absence or presence of the longer tethers [29,38,39,40].

In comparison to the open form (*R*-**BNPB**), the *cisoid* conformation in the bridged *R*-**7BBNPB** leads to 3-fold enhancement of the |*g*_abs_| value from 6.0 × 10^–4^ to 1.7 × 10^–3^. However, when the number of bridging carbon atoms is increased from one to two, the |*g*_abs_| is reduced to 8.8 × 10^−4^ owing to a more flexible structure. As for the green-yellow emitters (*R*-**BNBTPB**, *R*-**7BBNBTPB**, and *R*-**BNCNCzPB**), they all have similar CD intensities with |*g*_abs_| values of ca. 10^–4^.

The observation of circular dichroism signals for all the chiral chromophores indicates that the chiral environment of the chromophore is coupled to the electronic transitions from the ground to the excited state. This in turn suggests that the luminescence of the excited state may also present chiroptical effects. The efficiency of the chiral induction can be quantified by the asymmetry factor (*g*_lum_) according to Equation (1) [41].

(1) $$g_{\mathrm{lum}}=2\frac{I_{L}\;-\;I_{R}}{I_{L}+I_{R}}$$

were *I*_L_ and *I*_R_ are the intensities of the left and right CPL, respectively.

**Figure 6:**
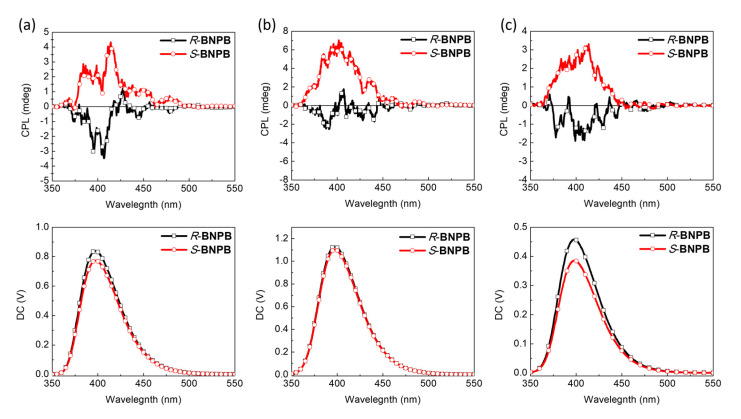
CPL spectra (λ_ex_ = 300 nm) of R- and S-**BNPB** in THF (**a**) 10^–4^ M (**b**) 10^–5^ M solutions and (**c**) toluene solutions (10^–5^ M).

The CPL spectra of the compounds were measured in different solvents and concentrations to find the optimal conditions for CPL emission in solution, possibly enhanced by the self-assembly of H-B nanostructures. As shown in Figure 6, **BNPB** shows distinct CPL in THF solutions with a mirror–image relationship for the two enantiomers. Upon diluting the concentration from 10^−4^ to 10^–5^ M, a stronger ellipticity and fluorescence intensity resulted in higher *g*_lum_ = 3.8 × 10^–4^ at 398 nm. A similar result was obtained in diluted THF/toluene solutions (1/9, *v/v*), albeit with a lower emission intensity.

**Figure 7:**
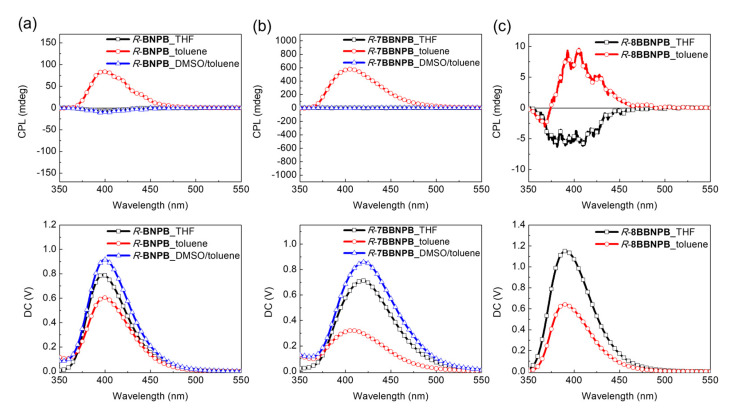
CPL spectra of (**a**) *R*-**BNPB**, (**b**) *R*-**7BBNPB**, and (**c**) *R*-**8BBNPB** in THF, toluene, or toluene/DMSO solutions. λ_ex_ = 300 nm.

In the case of **BNBTPB** and **7BBNBTPB**, no significant CPL was detected from either enantiomer regardless of the solvent (THF or toluene solution) or concentration (see Figures S1 and S2). The situation is similar for **BNCNCzPB**, for which the CPL spectra is very weak. In contrast, an unusually large asymmetry factor was observed for *R*-**7BBNPB** which could not be reproduced for the S enantiomer. Examination of the linear dichroism signal did not reveal strong contributions from linear polarization which may induce artefacts in the determination of the *g*_lum_ value. On the other hand, aggregation has been shown to strongly amplify the asymmetry factor of small molecules. To verify whether self-assembly of the aggregates contributes to the observed enhancement of the g_lum_ value, DMSO was added to break up the intermolecular hydrogen-bonding network and induce the dissolution of the vesicles. This was indeed found to lead to the disappearance of the CPL signal, in agreement with the enhancement of the asymmetry due to aggregation (Figure 7).

## 3. Discussion

The emissive systems composed of axially chiral binaphthalenes appended with H-B biuret motifs were found to spontaneously form nanospheres whose size ranged from ca. 250 to 500 nm diameter depending on the environment (concentration, solvent composition). It is interesting to note that, for each compound, the average dimensions of the spherical aggregates are not significantly dependent on the solvent system. This suggests that the formation of the aggregates is mostly determined by the molecular structure rather than solvent–solute interactions as is the case for other amphiphilic systems. The boundary conditions are met when the solubility is insufficient (precipitation) or too high (formation of amorphous films). The aggregates show tunable emission from deep-blue to yellow through the introduction of benzothiadiazole groups onto the π-conjugated backbone. As expected, all the chromophores exhibit intense Cotton effects with mirror–image relationship between the enantiomers in the ECD spectra, indicating chiroptical activity in the ground state. For **BNCNCzPB**, a possible CPL-TADF emitter, the steady-state and time-resolved PL spectra at variable temperature show that the TADF character is absent. Examination of the phosphorescence spectra of the parent **BNPB** chromophore reveals that the energy level of the triplet state is located at ca. 2.30 eV, which is much too far below the energy of the singlet state (3.24 eV) to expect efficient ISC. Thus, the π-conjugation in the **BNCNCzPB** fluorophore likely leads to an increase in Δ*E*_ST_ that turns off the ISC channel needed to harvest triplet excitons. This behavior is in agreement with an extension of the conjugation of the chromophore due to the presence of the phenyl-biuret moieties, which leads to a decrease in the energy of the excited singlet and triplet states with respect to that of the ground state. In the case of **BNCNCzPB**, the electron-withdrawing nature of the biuret further lowers the energy of the triplet state by reducing its CT character, thus explaining the increased Δ*E*_ST_.

From the preliminary results collected for *R*-**BNPB**, *R*-**7BBNPB**, and *R*-**8BBNPB**, it is possible to observe moderate CPL intensity and inversion of signal in toluene solutions (5 × 10^–5^ M) compared to THF (Figure 7). In Equation (2), it can be seen that the magnitude and angle between the electric transition dipole moment (***μ***) and the magnetic transition dipole moment (***m***) significantly influence the *g*_lum_ value [41], and we may surmise that the modest value of *g*_lum_ results from a large value of |***µ***| in agreement with the high *F*_F_ of these compounds.

(2) $$g_{\mathrm{lum}}=4\times \frac{\left|m\right|}{\left|\mu \right|}\;\cos\theta_{\mu ,\;m}$$

Remarkably, for *R*-**BNPB** in the open form, the *g*_lum_ value is increased from 10^4^ in THF to ca. 10^–2^ in toluene solution as a result of aggregation. Unfortunately, this could not be reproducibly confirmed for the S-form enantiomer. Considering that the dissymmetry factor appears to be highly dependent on the aggregation conditions, we may expect that small differences in samples may explain this behavior. Further investigation of the photophysical and chiroptical properties of **BNPB** and **7BBNPB** in solution and in the solid state is needed to understand which parameters govern chiral amplification through aggregate formation in these assemblies. For the group of deep-blue emitters, the *g*_abs_ value of the bridged *R*-**7BBNPB** (1.7 × 10^–3^) in the restrained *cisoid* conformation is 3-fold higher than the one of the unrestrained form *R*-**BNPB** (6.0 × 10^–4^). From preliminary CPL results, the structural enforcement of the *cisoid* conformation in *R*-**7BBNPB** induces a positive effect on the amplification of the asymmetry factor through aggregation which disappears upon addition of DMSO to break up the aggregates.

## 4. Materials and Methods

All the starting materials and solvents were purchased from commercial sources and used without further purification unless stated otherwise. ^1^H and ^13^C NMR spectra of compounds were collected on a Varian 400 Unity plus (400 MHz) spectrometer in deuterated solvents as internal reference at room temperature. Mass spectra were recorded using a Bruker micrOTOF-QII mass spectrometer and Bruker Daltonics autoflex speed with electrospray ionization (ESI) and matrix-assisted laser desorption/ionization (MALDI) as ionization method. The mass spectrum was recorded with JEOL JMS-700 with electron ionization (EI). UV-visible absorption spectra were recorded on a spectrophotometer (HITACHI U2800A) or Perkin-Elmer 750 spectrophotometer. Emission spectra were measured with a fluorescence spectrophotometer (HITACHI F9500) or a Horiba Fluoromax 4 instrument. Photoluminescence quantum yields (PLQYs) for solution and thin-film samples were determined with a calibrated integrating sphere system (HAMAMATSU C9920). The circular dichroism (CD) spectra were measured on a JASCO J-810 circular dichroism spectrometer with ‘Standard’ sensitivity. The scan speed was set as 200 nm/min with a 0.1 nm resolution and a response time of 2.0 s. The circularly polarized photoluminescence (CP-PL) spectra were measured on a JASCO CPL-300 spectrophotometer with ‘Standard’ sensitivity at 100 nm/min scan speed and a response time of 4.0 s employing the “slit” mode.

### 4.1. Preparation of Nanospheres

Solutions of compounds were prepared by adding the appropriate volume of THF (dried over Na/ benzophenone and distilled prior to use), acetone (ACS reagent, 99.5+%, for analysis), or methanol (ACS reagent, 99.5+%, for analysis) to a glass vial containing a solid sample of the compound to produce a 0.1 mM solution. Dissolution was achieved by gentle shaking of the vial for 30 min. The solutions were drop-casted onto the substrates using a syringe to deposit approximately 50 μL of solution, and the solvent was allowed to evaporate in air.

### 4.2. Techniques for Characterization

Scanning Electron Microscopy. Samples were prepared by drop-casting a THF or acetone or methanol solution of self-assembled compounds (0.1 mM) onto a flat SiO_2_/Si substrate as described above. The samples were imaged using a FEI Nova NanoSEM 200 in low-vacuum mode with no conductive overcoat. The chamber pressure was maintained at 0.45 Torr water using a differential pumping system. An immersion lens was employed, and the secondary electrons were amplified by gas vapor and collected by an electrode mounted on the pole piece.

Variable-Temperature PL spectra and Time-Correlated Single Photon Counting. Decay parameters were extracted from reconvolution of the time-resolved emission decay profiles collected using a home-built TC-SPC instrument equipped with a Picoquant 310 nm or 350 nm pulsed excitation source and a Hamamatsu R6427 photomultiplier and Timeharp 260 collection electronics. A monochromator or 420 long-pass filter was used to isolate the emission from the sample. Variable-temperature spectra and lifetimes were obtained using an Oxford DN optistat connected to an ITC controller (precision = ±0.1 K).

## 5. Conclusions

The incorporation of H-B biuret elements to chiral, rigid chromophores is found to be an efficient promoter for the formation of small, sub-micrometer vesicle-like aggregates. These are formed in various organic solvents such as toluene, acetone, or THF. Nonetheless, extension of the π-conjugation of the chromophore through the introduction of the phenylbiuret units can have unforeseen consequences, such as increasing the ∆*E*_ST_ splitting in TADF chromophores. In all cases, the chiral component of the binaphthalene core was found to impart chiroptical properties to the chromophores, particularly in the ECD spectra of the ground state. In contrast, the CPL activity is moderate or absent, presumably due to a planarization of the chromophore in the excited state. When this is impeded by a short, rigid linker, an increase in the emission asymmetry factor is observed. Interestingly, we find that, for the R enantiomer, amplification of the asymmetry factor is observed upon aggregation. The fact that this is not observed for the S enantiomer suggests that there are subtle differences in the sample composition or aggregate formation that need to be uncovered to fully understand how self-assembly affects supramolecular chiral induction.

## Data Availability

Experimental data (electronic and emission spectra, NMR data) are available from the authors upon reasonable request.

## Supplementary Materials

The following supporting information can be downloaded at: https://www.mdpi.com/article/10.3390/molecules28083382/s1, Details of synthesis, additional CPL spectra.

## Sample Availability

Samples of the compounds used in this study are available from the authors upon reasonable request and if in stock.
