# Supplementary material for: Chiral Binaphthalene Building Blocks for Self-Assembled Nanoscale CPL Emitters
Source: Molecules. 2023 Apr 11;28(8):3382. doi: 10.3390/molecules28083382 (PMC10142354; doi:10.3390/molecules28083382)
Supplement: Supplementary file 1 [file molecules-28-03382-s001.zip › molecules-2326422-supplementary.pdf]

Supporting Information

Chiral Binaphthalene Building Blocks for Self-Assembled Nanoscale CPL Emitters

Yu-Yu Hsieh [1,2], Jing-Jong Shyue [3], Ken-Tsung Wong [2,4]* and Dario M. Bassani [1,]*

1 Univ. Bordeaux, CNRS, Bordeaux INP, ISM UMR 5255, F-33400 Talence, France.
2 Department of Chemistry, National Taiwan University, Taipei, 10617, Taiwan.
3 Research Center for Applied Sciences, Academia Sinica, Taipei 11529, Taiwan.
4 Institute of Atomic and Molecular Science, Academia Sinica, Taipei 10617, Taiwan.
* Correspondence: dario.bassani@u-bordeaux.fr (DMB); kenwong@ntu.edu.tw (KTW)

1. Synthesis

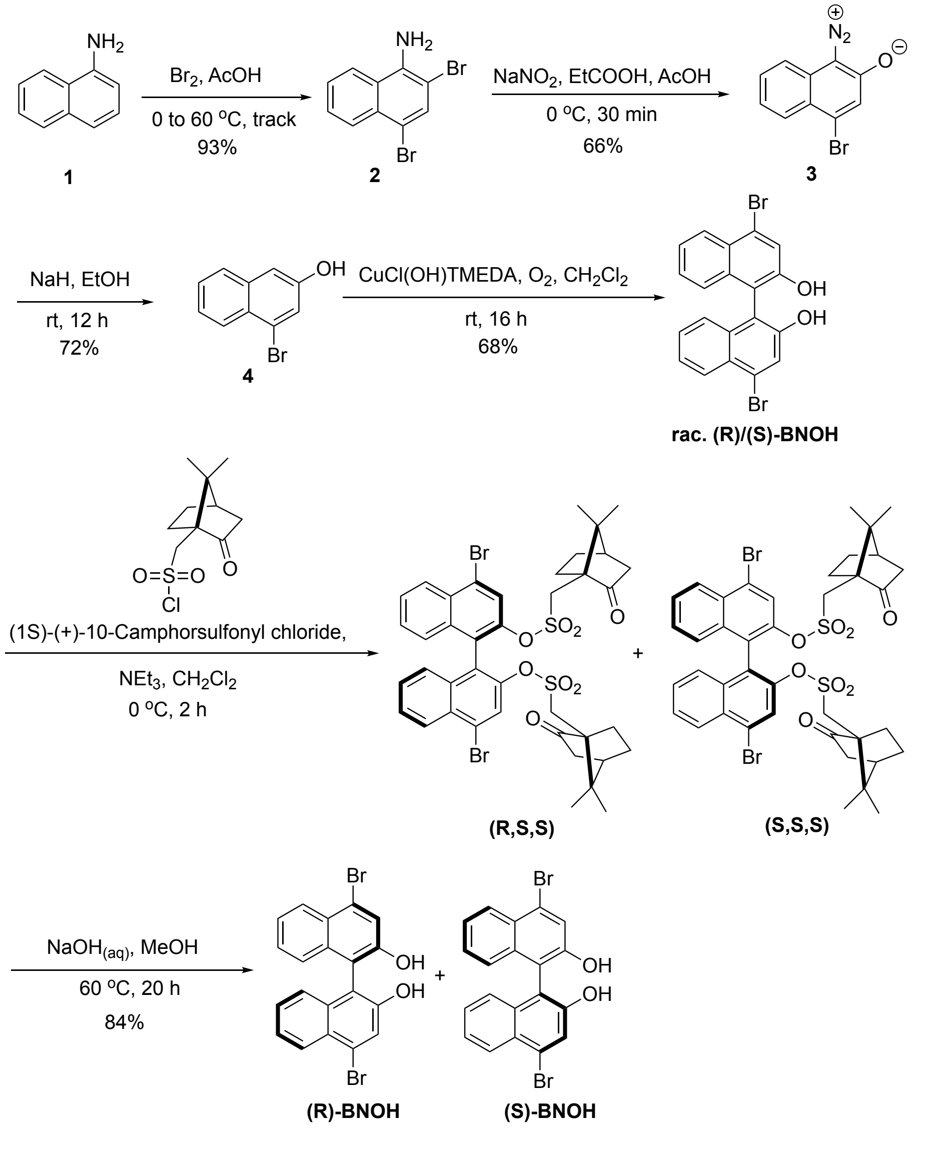

Scheme S1. Synthetic route for the preparation of BNOH [1].

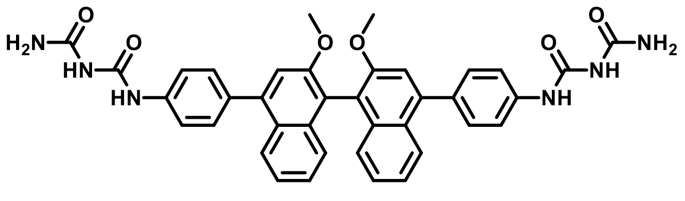

BNPB

Synthesis of **BNPB**. A mixture of $Pd(PPh_3)_4$ (49 mg, 0.042 mmol), K_2CO_3 (351 mg, 2.54 mmol), **BNOMe** (200 mg, 0.42 mmol), 4-pinacolatoboronic ester-benzenebiuret (271 mg, 0.89 mmol) in degassed THF (4.5 mL) and water (1.3 mL) was refluxed for two days. The reaction was quenched by adding water and extracted with THF. The combined organic solution was washed with brine and dried over $MgSO_4$. The crude product was purified by column chromatography on silica gel (THF/CH_2Cl_2 = 1/1) to afford **BNPB** (55%, 155 mg) as a white solid. ^{1}H NMR (d_6-DMSO, 400 MHz) δ 10.14 (s, 2H), 8.95 (s, 2H), 7.85 (d, J = 3.8 Hz, 2H), 7.66 (d, J = 4.0 Hz, 2H), 7.59 (d, J = 4.0 Hz, 2H), 7.47 (s, 2H), 7.27 (quint, J = 6.8 Hz, 4H), 7.04 (d, J = 4.0 Hz, 2H), 6.95 (bs, 4H), 3.76 (s, 6H); ^{13}C NMR (d_6-DMSO, 100 MHz) δ 155.5, 154.1, 152.1, 140.7, 137.7, 134.6, 133.9, 130.6, 126.6, 126.4, 125.7, 125.1, 123.6, 119.1, 117.9, 115.0, 56.2; HRMS (m/z, MALDI) Calcd for $C_{38}H_{32}N_6O_6$ 668.2383; found 668.2398.

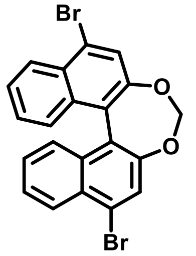

7BBNBr2

Synthesis of **7BBNBr2**. A mixture of **BNOH** (820 mg, 1.84 mmol), K_2CO_3 (2.55 g, 18.5 mmol) in dried DMF (61.5 mL) were stirred in argon atmosphere at 50 °C for 15 min, followed by addition of bromochloromethane (3.72 mL, 55.2 mmol) dropwise and stirred in argon atmosphere at 50 °C for 15 h. The reaction was quenched by adding water and brine and extracted by ethyl acetate. The combined organic phase was dried over $MgSO_4$. The crude product was purified by column chromatography on silica gel (hexanes /CH_2Cl_2 = 4/1) to afford **7BBNBr2** (86%, 725 mg) as a white solid. ^{1}H NMR ($CDCl_3$, 400 MHz) δ 8.36 (d, J = 4.4 Hz, 2H), 7.82 (s, 2H), 7.58-7.54 (m, 2H), 7.45 (d, J = 4.0 Hz), 7.36-7.32 (m, 2H), 5.68 (s, 2H); ^{13}C NMR ($CDCl_3$, 100 MHz) δ 132.6, 130.3, 127.6, 127.1, 127.0, 126.5, 125.5, 125.2, 124.9, 124.0, 121.5, 103.3; HRMS (m/z, MALDI) Calcd for $C_{21}H_{12}{}^{79}Br_2O_2$ 453.9198; found 453.9177; Calcd for $C_{21}H_{12}{}^{79}Br^{81}BrO_2$ 455.9179; found 455.9190; Calcd for $C_{21}H_{12}{}^{81}Br_2O_2$ 457.9162; found 457.9209;

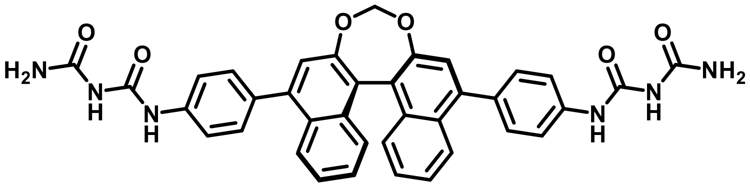

7BBNPB

Synthesis of **7BBNPB**. A mixture of $Pd(PPh_3)_4$ (30 mg, 0.026 mmol), K_2CO_3 (447 mg, 2.99 mmol), **7BBNBr2** (228 mg, 0.50 mmol), 4-pinacolatoboronic ester-benzenebiuret (335 mg, 1.10 mmol) in degassed THF (25 mL) and water (1.5 mL) was refluxed for two days. The reaction was quenched by adding water and extracted with THF. The combined organic solution was washed with brine and dried over $MgSO_4$. The crude product was purified by column chromatography on silica gel (THF/CH_2Cl_2 = 1/1) to afford **7BBNPB** (63%, 205 mg) as a white solid. ^1H NMR (d_6-DMSO, 400 MHz) δ 10.02 (s, 2H), 8.97 (s, 2H), 7.99 (d, J = 4.4 Hz, 2H), 7.68 (d, J = 4.0 Hz, 2H), 7.59 (d, J = 4.0 Hz, 2H), 7.50-7.48 (m, 6H), 7.43 (d, J = 3.6 Hz, 2H), 6.95 (bs, 4H), 5.78 (s, 2H); ^{13}C NMR (d_6-DMSO, 100 MHz) δ 155.5, 152.1, 150.6, 141.8, 138.0, 133.8, 132.0, 130.5, 129.3, 126.5, 126.4, 125.5, 124.5, 121.9, 119.1, 103.0; HRMS (m/z, MALDI) Calcd for $C_{37}H_{28}N_6O_6$ 652.2070; found 652.2033.

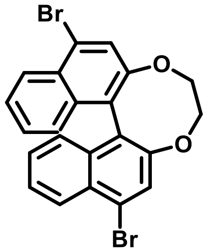

8BBNBr2

Synthesis of **8BBNBr2**. A mixture of **BNOH** (444 mg, 1.00 mmol), K_2CO_3 (1.03 g, 7.49 mmol) and ethylene glycol ditosylate (462 mg, 1.25 mmol) in dried DMF (13 mL) were stirred in argon atmosphere at 80 °C for 18 h. The reaction was quenched by adding water and brine and extracted by ethyl acetate. The combined organic phase was dried over $MgSO_4$. The crude product was purified by column chromatography on silica gel (hexanes/CH_2Cl_2 = 4/1 to 2/1) to afford **8BBNBr2** (50%, 235 mg) as a white solid. ^1H NMR (CD_2Cl_2, 400 MHz) δ 8.31 (d, J = 4.6 Hz, 2H), 7.82 (s, 2H), 7.53 (t, J = 6.4 Hz, 2H), 7.28 (t, J = 7.2 Hz, 2H), 7.20 (d, J = 4.2 Hz, 2H), 4.41 (q, J = 9.2 Hz, 2H), 4.16 (q, J = 8.8 Hz, 2H); ^{13}C NMR (CD_2Cl_2, 100 MHz) δ 156.7, 134.0, 130.1, 127.8, 127.8, 127.7, 127.4, 126.9, 124.7, 124.3, 73.8; HRMS (m/z, MALDI) Calcd for $C_{22}H_{14}{}^{79}Br_2O_2$ 467.9355; found 467.9375; Calcd for $C_{22}H_{14}{}^{79}Br^{81}BrO_2$ 469.9335; found 469.9378; Calcd for $C_{22}H_{14}{}^{81}Br_2O_2$ 471.9319; found 471.9376.

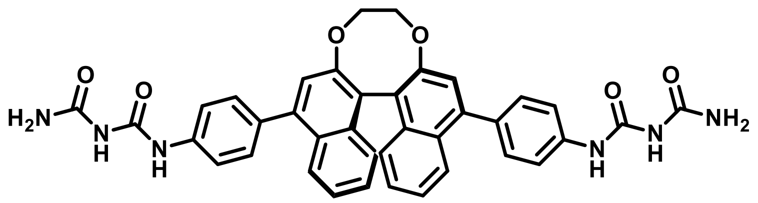

8BBNPB

Synthesis of **8BBNPB**. A mixture of $Pd(PPh_3)_4$ (6.5 mg, 0.0056 mmol), K_2CO_3 (93 mg, 0.67 mmol), **8BBNBr2** (53 mg, 0.11 mmol), 4-pinacolatoboronic ester-benzenebiuret (76 mg, 0.25 mmol) in degassed THF (5.0 mL) and water (0.3 mL) was refluxed for two days. The reaction was quenched by adding water and extracted with THF. The combined organic solution was washed with brine and dried over $MgSO_4$. The crude product was purified by column chromatography on silica gel (THF/CH_2Cl_2 = 1/1) to afford **8BBNPB** (28%, 21 mg) as a white solid. ^1H NMR (d_6-DMSO, 400 MHz) δ 10.15 (s, 2H), 8.95 (s, 2H), 7.92 (d, J = 4.4 Hz, 2H), 7.65 (d, J = 4.2 Hz, 2H), 7.58 (d, J = 4.4 Hz, 2H), 7.43 (s, 2H), 7.40 (d, J = 4.2 Hz, 2H), 7.32 (t, J = 7.2 Hz, 2H), 7.23 (d, J = 4.4 Hz, 2H), 6.95 (bs, 4H), 4.41 (d, J = 4.4 Hz, 2H), 4.15 (d, J = 4.4 Hz, 2H); ^{13}C NMR (d_6-DMSO, 100 MHz) δ 155.9, 155.5, 152.1, 142.1, 137.9, 134.0, 132.9, 130.5, 128.5, 126.8, 126.5, 125.9, 125.1, 123.5, 122.9, 119.1, 72.9; HRMS (m/z, MALDI) Calcd for $C_{38}H_{30}N_6O_6$ 667.2299; found 667.2268 (M+1).

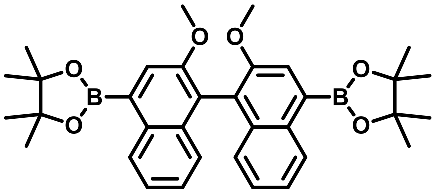

BNBpin2

Synthesis of **BNBpin2**. A mixture of **BNOMe** (100 mg, 0.21 mmol), bis(pinacolato)diborane (162 mg, 0.63 mmol), KOAc (125 mg, 1.26 mmol) and $Pd(dppf)Cl_2$ (14 mg, 0.02 mmol) in dried THF (5 mL) were stirred in argon atmosphere at 80 °C for 18 h. The reaction was cooled and concentrated in vacuo to get crude. The crude product was purified by column chromatography on silica gel (hexanes/THF = 4/1) to afford **BNBpin2** (82%, 97 mg) as white crystals. ^1H NMR (d_6-DMSO, 400 MHz) δ 8.70 (d, J = 4.4 Hz, 2H), 7.93 (s, 2H), 7.38 (t, J = 8.0 Hz, 2H), 7.21 (t, J = 8.0 Hz, 2H), 6.91 (d, J = 4.0 Hz, 2H), 3.72 (s, 6H), 1.43 (s, 24H); ^{13}C NMR (d_6-DMSO, 100 MHz) δ 153.5, 133.3, 132.1, 128.6, 128.1, 126.2, 124.8, 124.0, 122.5, 122.3, 83.9, 56.2, 24.7; HRMS (m/z, MALDI) Calcd for $C_{34}H_{40}B_2O_6$ 566.3011; found 566.3035.

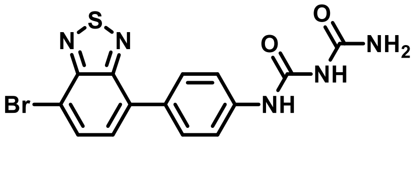

BTBiuret

Synthesis of **BTBiuret**. A mixture of $Pd(PPh_3)_4$ (37.8 mg, 0.033 mmol), K_2CO_3 (271 mg, 1.96 mmol), **BTBr2** (385 mg, 1.31 mmol), 4-pinacolatoboronic ester-benzenebiuret (200 mg, 0.65 mmol) in degassed THF (26 mL) and water (1.0 mL) was refluxed for two days. The reaction was quenched by adding water and extracted with THF and saturated $NH_4Cl_{(aq)}$. The combined organic solution was washed with brine and dried over $MgSO_4$. The crude product was purified by column chromatography on silica gel (THF/ hexanes = 1/1 to 3/1) to afford **BTBiuret** (56%, 290 mg) as a yellow solid. ^1H NMR (d_6-DMSO, 400 MHz) δ 10.18 (s, 2H), 8.95 (s, 2H), 8.10 (d, J = 3.8 Hz, 2H), 7.95 (d, J = 4.4 Hz, 4H), 7.76 (d, J = 3.8 Hz, 2H), 7.63 (d, J = 4.2 Hz, 4H), 6.90 (bs, 4H); ^{13}C NMR (d_6-DMSO, 100 MHz) δ 165.1, 162.8, 162.1, 161.6, 148.3, 142.2, 142.0, 140.3, 139.4, 137.6, 128.6, 121.3.

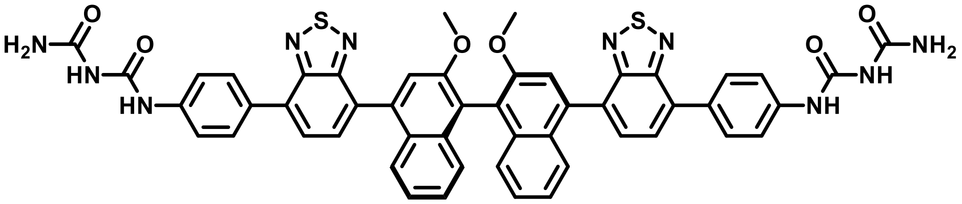

BNBTPB

Synthesis of **BNBTPB**. A mixture of $Pd(PPh_3)_4$ (20 mg, 0.010 mmol), K_2CO_3 (146 mg, 1.05 mmol), **BTBiuret** (148 mg, 0.37 mmol), **BNBpin2** (100 mg, 0.18 mmol) in degassed THF (7.0 mL) and water (0.5 mL) was refluxed for two days. The reaction was quenched by adding water and extracted with THF. The combined organic solution was washed with brine and dried over $MgSO_4$. The crude product was purified by column chromatography on silica gel (THF/CH_2Cl_2 = 1/1) to afford **BNBTPB** (45%, 74 mg) as a yellow solid. ^1H NMR (d_6-DMSO, 400 MHz) δ 10.20 (s, 2H), 8.97 (s, 2H), 8.09 (d, J = 4.2 Hz, 4H), 8.03 (q, J = 7.2 Hz, 4H), 7.77 (s, 2H), 7.68 (d, J = 4.4 Hz, 4H), 7.56 (d, J = 4.2 Hz, 2H), 7.30 (t, J = 7.2 Hz, 2H), 7.18 (t, J = 6.0 Hz, 2H), 7.13 (d, J = 6.0 Hz, 2H), 6.90 (bs, 4H), 3.79 (s, 6H); ^{13}C NMR (d_6-DMSO, 100 MHz) δ 155.5, 154.8, 154.1, 152.9, 152.1, 138.5, 137.1, 133.7, 132.4, 131.8, 131.5, 131.0, 129.8, 128.9, 128.2, 127.4, 127.2, 126.5, 123.7, 119.1, 118.9, 116.2, 56.3; HRMS (m/z, MALDI) Calcd for $C_{50}H_{36}N_{10}O_6S_2$ 936.2261; found 936.2278.

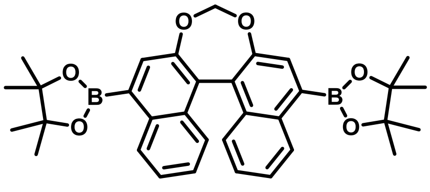

7BBNBpin2

Synthesis of **7BBNBpin2**. A mixture of **7BBNBr2** (228 mg, 0.50 mmol), bis(pinacolato)diborane (381 mg, 1.50 mmol), KOAc (294 mg, 3.00 mmol) and Pd(dppf)Cl_2 (70 mg, 0.10 mmol) in dried THF (10 mL) were stirred in argon atmosphere at 80 °C for 18 h. The reaction was cooled and concentrated in vacuo to get crude. The crude product was purified by column chromatography on silica gel (hexanes/THF = 3/1) to afford **7BBNBpin2** (84%,

230 mg) as white crystals. ^{1}H NMR (d_6-DMSO, 400 MHz) δ 8.78 (d, *J* = 4.4 Hz, 2H), 7.93 (s, 2H), 7.55 (t, *J* = 8.0 Hz, 2H), 7.34 (quin, *J* = 8.0 Hz, 4H), 5.72 (s, 2H), 1.22 (s, 24H); ^{13}C NMR (d_6-DMSO, 100 MHz) δ 150.2, 134.5, 131.5, 129.8, 128.7, 128.5, 126.3, 126.2, 125.7, 103.0, 84.1, 82.8, 81.4, 24.8; HRMS (m/z, MALDI) Calcd for $C_{33}H_{36}B_2O_6$ 550.2698; found 550.2722.

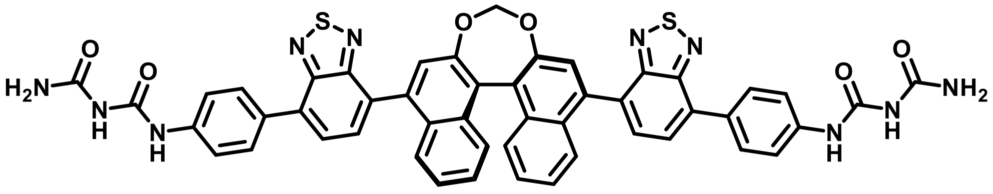

7BBNBTPB

Synthesis of **7BBNBTPB**. A mixture of $Pd(PPh_3)_4$ (19 mg, 0.016 mmol), K_2CO_3 (136 mg, 0.98 mmol), **BTBiuret** (137 mg, 0.34 mmol), **7BBNBpin2** (90 mg, 0.16 mmol) in degassed THF (7.0 mL) and water (0.5 mL) was refluxed for two days. The reaction was quenched by adding water and extracted with THF. The combined organic solution was washed with brine and dried over $MgSO_4$. The crude product was purified by column chromatography on silica gel (THF/CH_2Cl_2 = 1/1) to afford **7BBNBTPB** (35%, 53 mg) as an orange solid. ^{1}H NMR (d_6-DMSO, 400 MHz) δ 10.22 (s, 2H), 8.99 (s, 2H), 8.12 (d, *J* = 3.8 Hz, 4H), 8.08-8.01 (m, 4H), 7.76 (s, 2H), 7.70 (d, *J* = 4.2 Hz, 4H), 7.60 (d, *J* = 4.0 Hz, 2H), 7.46-7.41 (m, 4H), 7.00 (bs, 4H), 5.83 (s, 2H); ^{13}C NMR (d_6-DMSO, 100 MHz) δ 155.5, 154.7, 152.9, 152.1, 150.6, 138.6, 138.3, 132.6, 131.8, 131.3, 131.0, 130.9, 129.9, 129.4, 127.4, 126.9, 126.6, 126.4, 125.7, 125.5, 125.3, 123.1, 119.0, 103.2; HRMS (m/z, MALDI) Calcd for $C_{49}H_{32}N_{10}O_6S_2$ 920.1948; found 921.2061 (M+1).

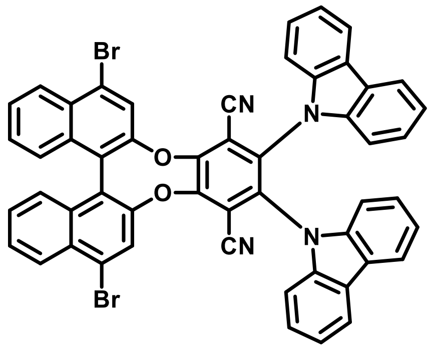

BNCNCzBr2

Synthesis of **BNCNCzBr2**. A mixture of **BNOH** (222 mg, 0.50 mmol), K_2CO_3 (138 mg, 1.00 mmol) and tetrafluoroterephthalonitrile (100 mg, 0.50 mmol) in dried DMF (2.5 mL) were stirred in argon atmosphere at room temperature for 12 h, followed by addition of K_2CO_3 (346 mg, 2.50 mmol) and carbazole (184 mg, 1.10 mmol) at room temperature for another 12 h. The solution was turned to in dark-green color with precipitates and quenched by adding water and extracted by CH_2Cl_2. The combined organic phase was dried over $MgSO_4$. The crude product was purified by column chromatography on silica gel (hexanes/ethyl acetate = 9/1) to afford

BNCNCzBr2 (40%, 180 mg) as a yellow solid. ^{1}H NMR (d_6-acetone, 400 MHz) δ 8.46 (d, *J* = 4.2 Hz, 2H), 8.14 (s, 2H), 7.85-7.80 (m, 4H), 7.77 (d, *J* = 4.2 Hz, 2H), 7.63 (d, *J* = 2.4 Hz, 4H), 7.46 (d, *J* = 4.0 Hz, 2H), 7.27 (d, *J* = 3.6 Hz, 2H), 7.20 (t, *J* = 7.6 Hz, 2H), 7.11 (t, *J* = 7.2 Hz, 2H), 6.98 (quint, *J* = 7.6 Hz, 4H); ^{13}C NMR (d_6-acetone, 100 MHz) δ 206.0, 152.2, 149.6, 140.6, 140.2, 137.2, 133.3, 131.9, 129.8, 129.4, 128.5, 128.1, 126.6, 125.8, 125.7, 124.7, 124.6, 121.9, 121.8, 121.0, 120.8, 116.1, 112.7, 111.5, 111.4, ; HRMS (m/z, MALDI) Calcd for $C_{52}H_{26}{}^{79}Br_2N_4O_2$ 896.0422; found 896.0312; Calcd for $C_{52}H_{26}{}^{79}Br^{81}BrN_4O_2$ 898.0403; found 898.0380; Calcd for $C_{52}H_{26}{}^{81}Br_2N_4O_2$ 900.0398; found 900.0386;

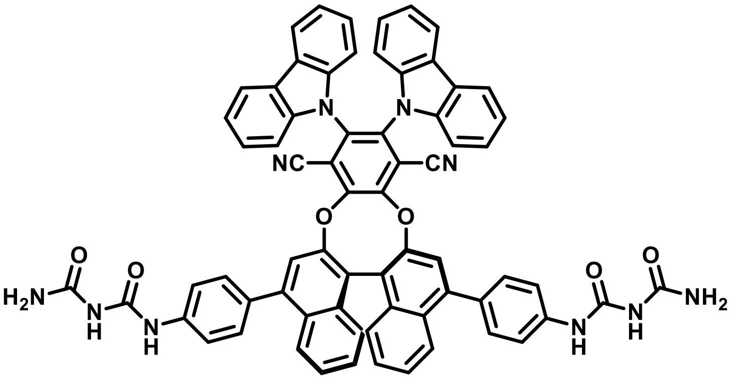

BNCNCzPB

Synthesis of **BNCNCzPB**. A mixture of $Pd(PPh_3)_4$ (10 mg, 0.0086 mmol), K_2CO_3 (83 mg, 0.60 mmol), **BNCNCzBr2** (90 mg, 0.10 mmol), 4-pinacolatoboronic ester-benzenebiuret (70 mg, 0.23 mmol) in degassed THF (3.0 mL) and water (0.3 mL) was refluxed for two days. The reaction was quenched by adding water and extracted with THF. The combined organic solution was washed with brine and dried over $MgSO_4$. The crude product was purified by column chromatography on silica gel (THF/CH_2Cl_2 = 1/2 to 1/1) to afford **BNCNCzPB** (67%, 74 mg) as a yellow solid. ^{1}H NMR (d_6-DMSO, 400 MHz) δ 10.22 (s, 2H), 8.99 (s, 2H), 8.14 (d, *J* = 4.4 Hz, 2H), 7.86 (t, *J* = 8.0 Hz, 4H), 7.74 (d, *J* = 2.2 Hz, 4H), 7.69 (m, 6H), 7.63 (s, 2H), 7.60 (d, *J* = 2.6 Hz, 4H), 7.54 (d, *J* = 4.0 Hz, 2H), 7.42 (d, *J* = 3.4 Hz, 2H), 7.15 (d, *J* = 4.0 Hz, 2H), 7.10-7.04 (m, 6H), 6.95 (bs, 4H); ^{13}C NMR (d_6-DMSO, 100 MHz) δ 155.4, 152.1, 151.3, 148.3, 143.2, 139.3, 139.2, 138.4, 135.6, 133.0, 132.0, 130.6, 130.3, 130.1, 127.9, 125.5, 123.3, 122.8, 121.4, 120.8, 120.1, 120.0, 119.6, 114.4, 112.1, 110.8, 110.7; HRMS (m/z, MALDI) Calcd for $C_{68}H_{42}N_{10}O_6$ 1094.3289; found 1095.3400 (M+1).

2. Circularly polarized luminescence spectra in solution

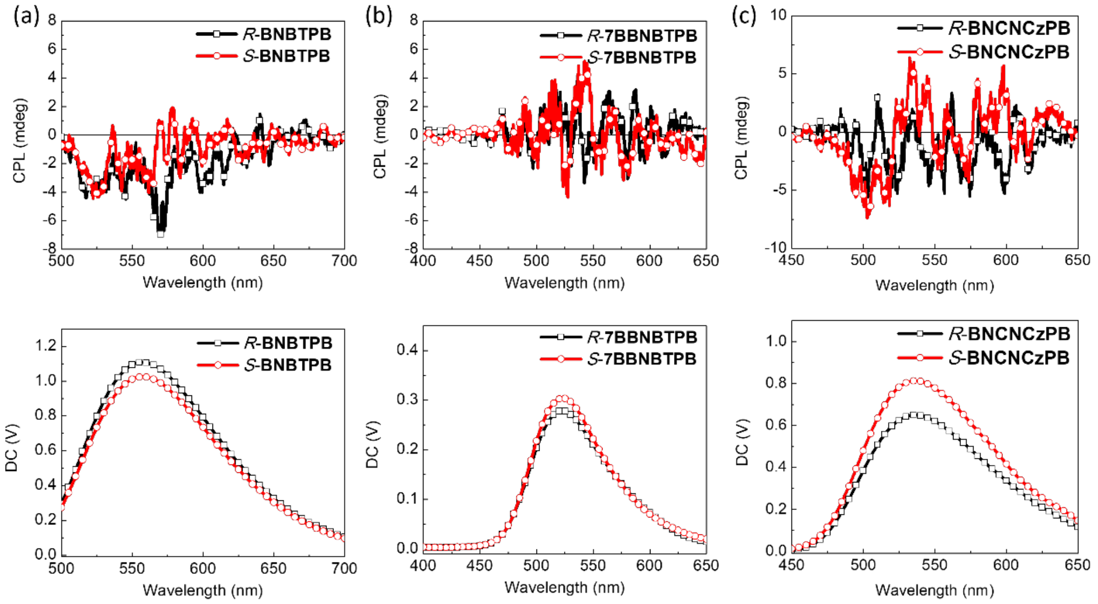

Figure S1. CPL spectra of (a) *R*/*S*-**BNBTPB**, (b) *R*/*S*-**7BBNBTPB** and (c) *R*/*S*-**BNCNCzPB** in THF solutions (10^{-5} M, λ_{ex} =300 nm).

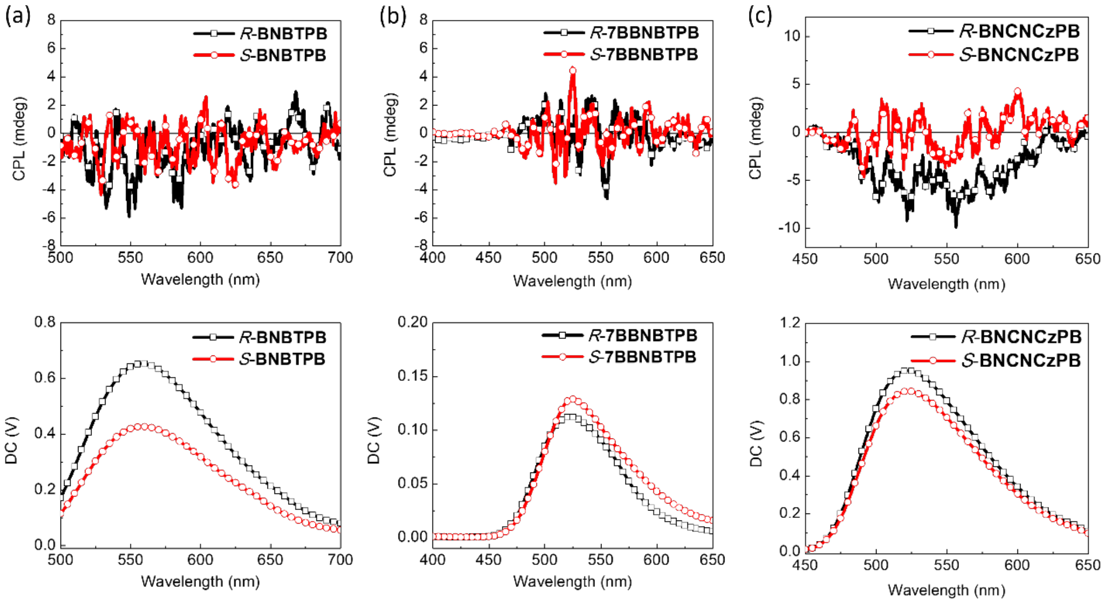

Figure S2. CPL spectra of (a) *R*/*S*-**BNBTPB**, (b) *R*/*S*-**7BBNBTPB** and (c) *R*/*S*-**BNCNCzPB** in toluene solutions (10^{-5}M, λ_{ex} =300 nm).

3. ^{1}H NMR and ^{13}C NMR spectra

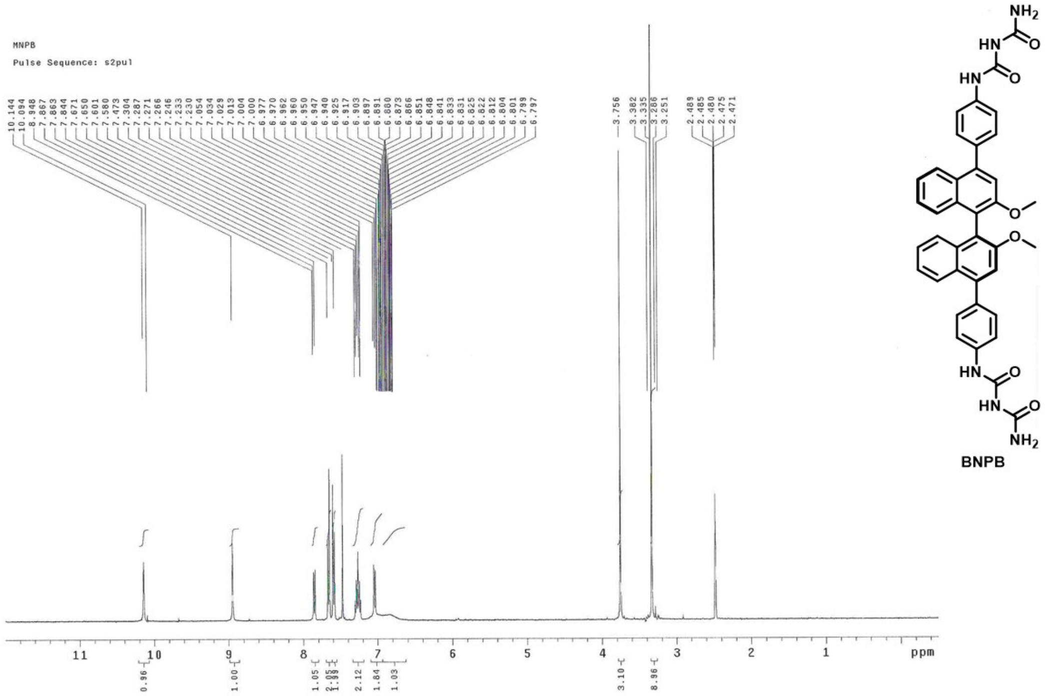

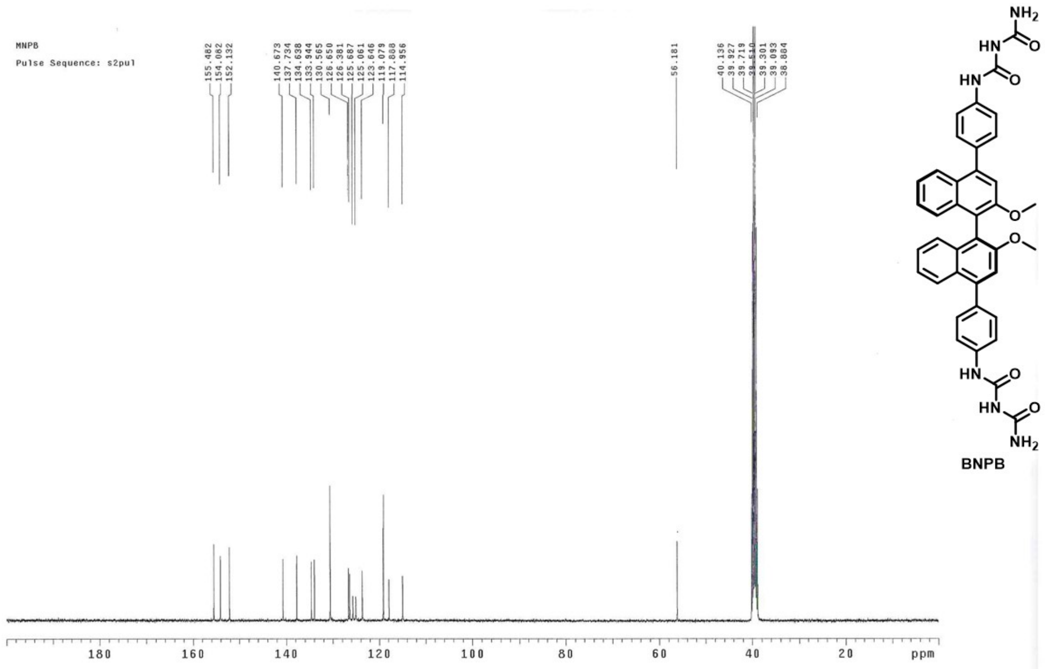

Figure S3. ^{1}H and ^{13}C spectra of compound **BNPB**.

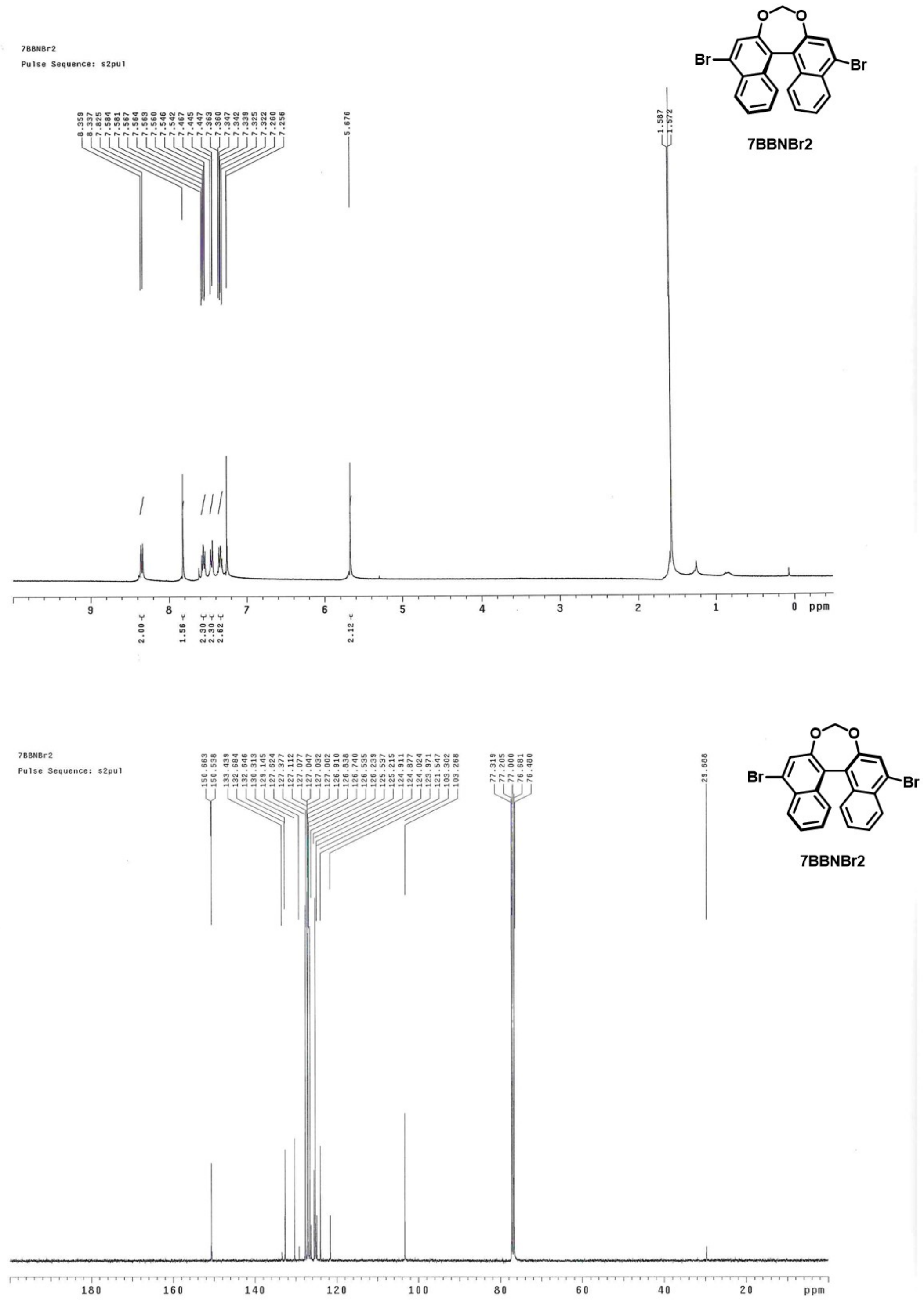

Figure S4. ^{1}H and ^{13}C spectra of compound **7BBNBr2**.

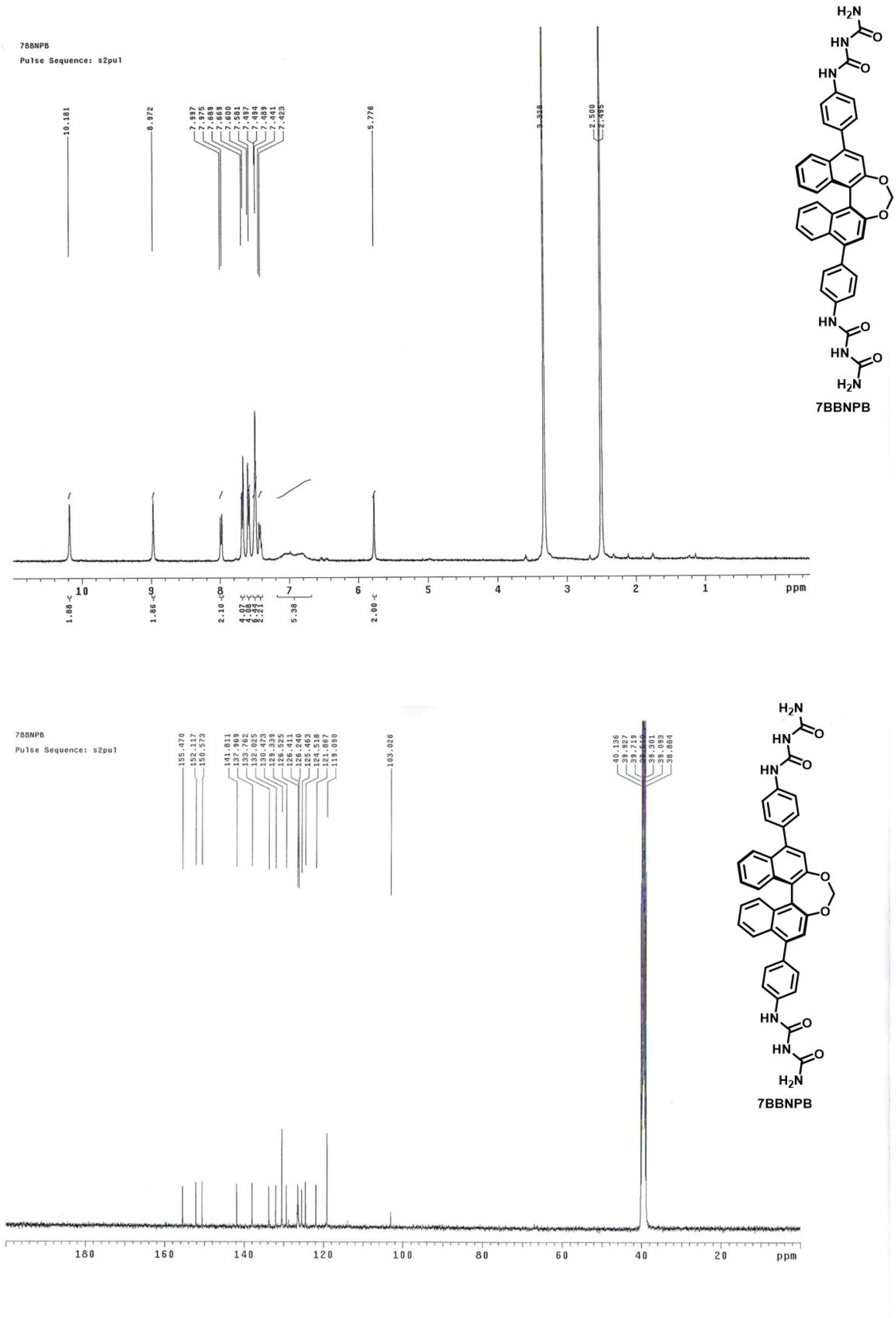

Figure S5. ^{1}H and ^{13}C spectra of compound **7BBNPB**.

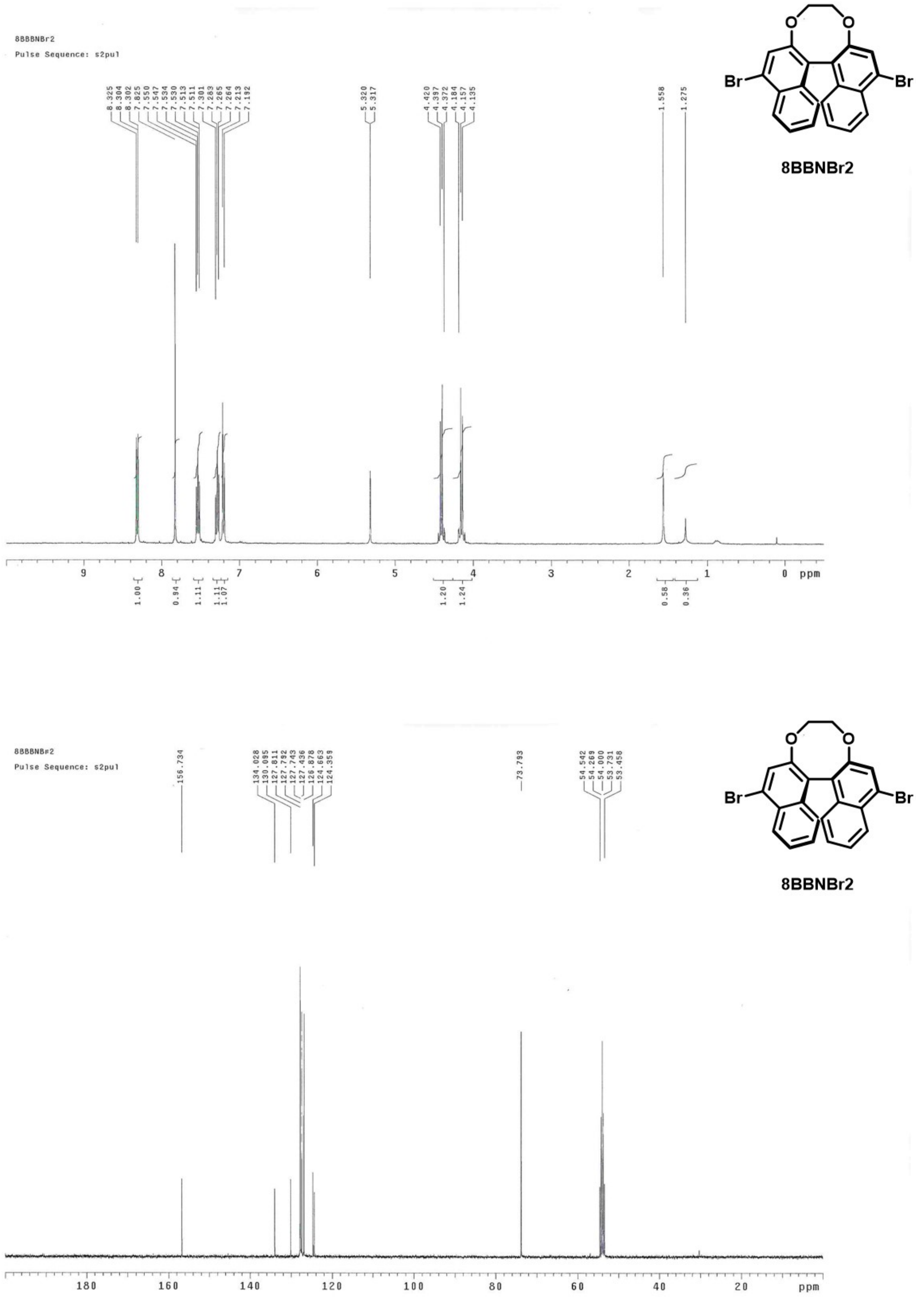

Figure S6. ^{1}H and ^{13}C spectra of compound **8BBNBr2**.

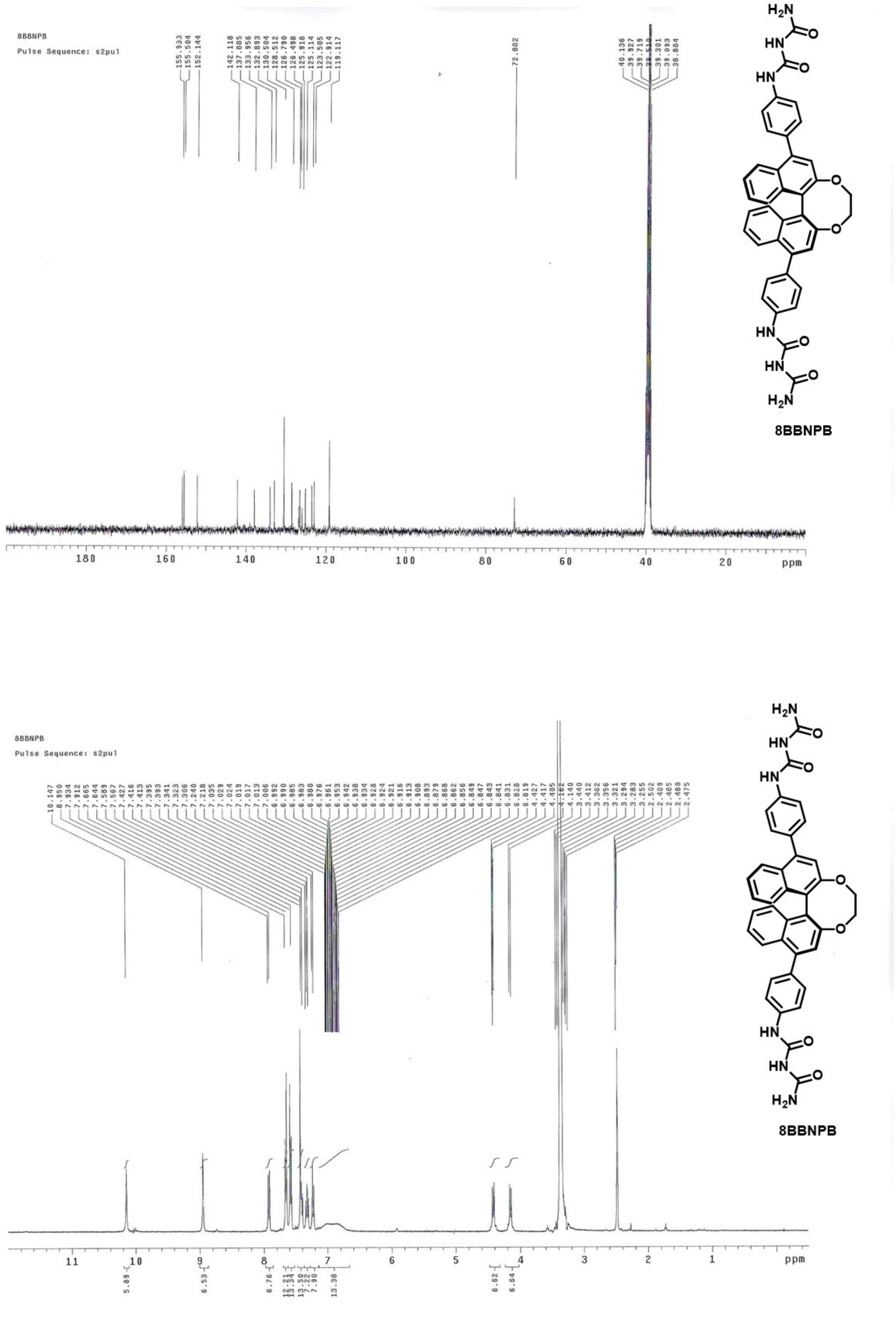

Figure S7. ^{1}H and ^{13}C spectra of compound **8BBNPB**.

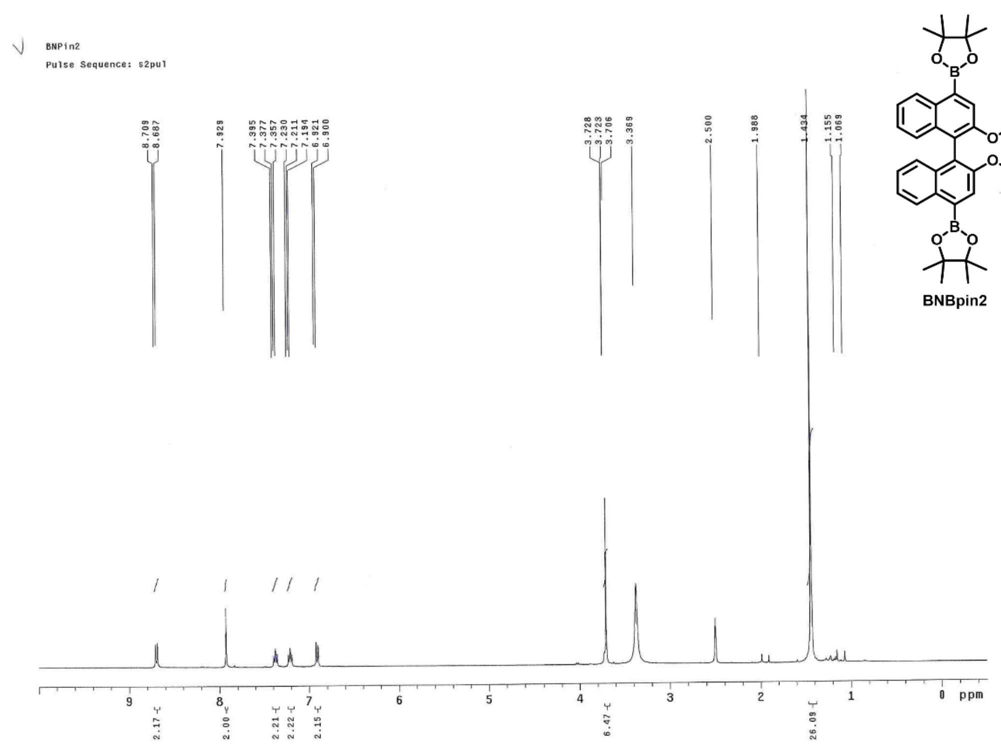

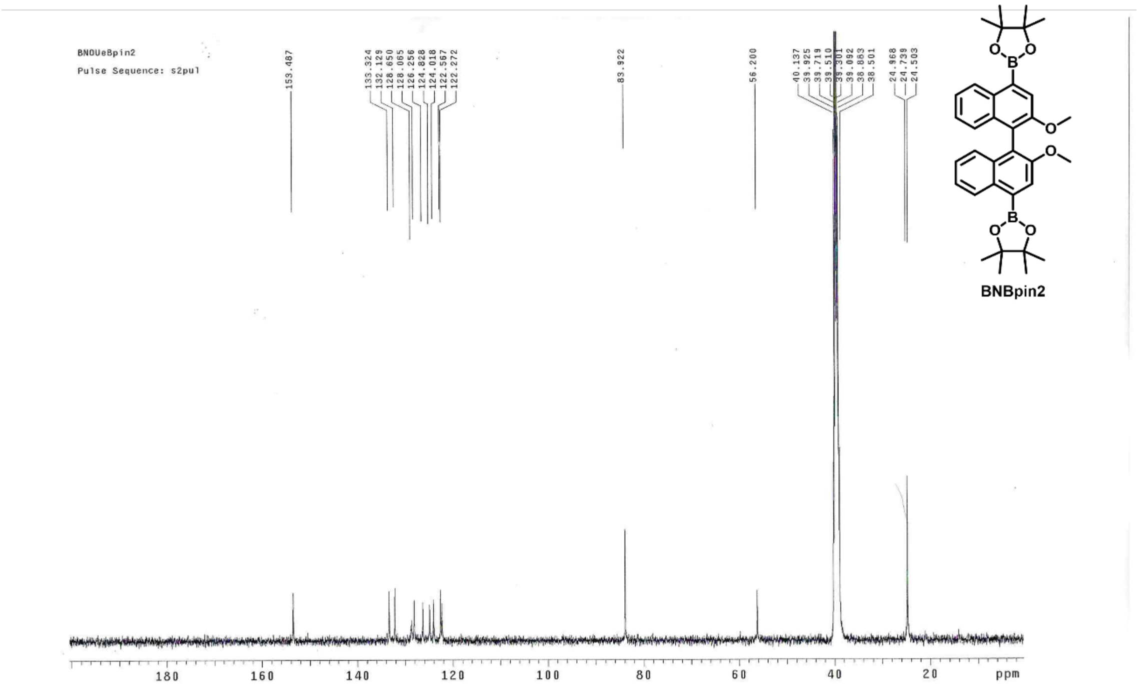

Figure S8. ^{1}H and ^{13}C spectra of compound **BNBpin2**.

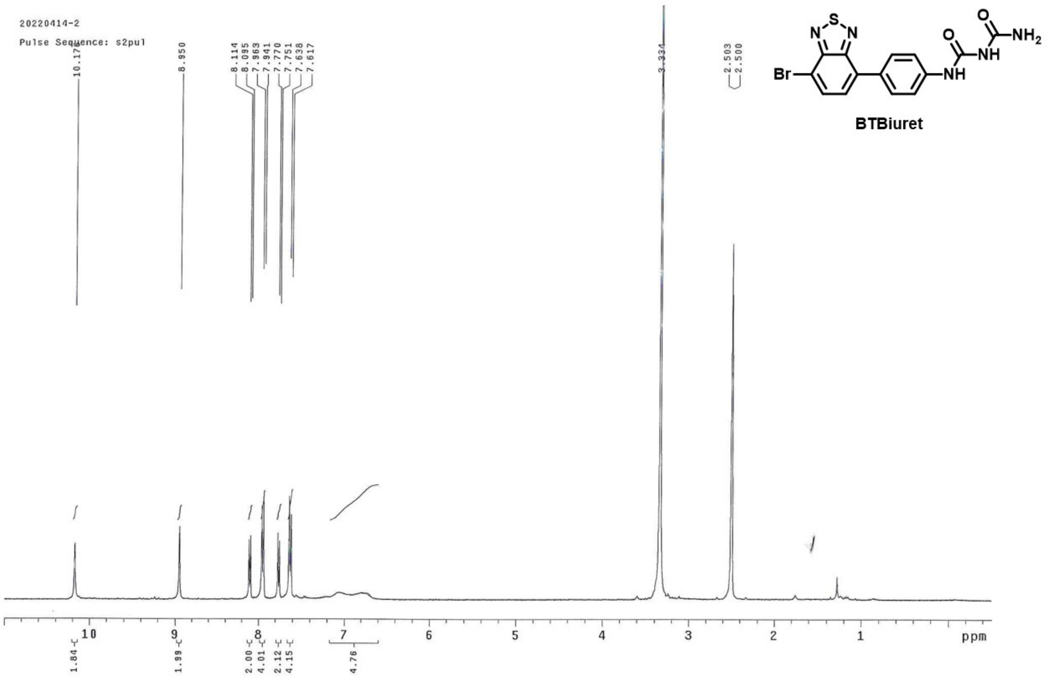

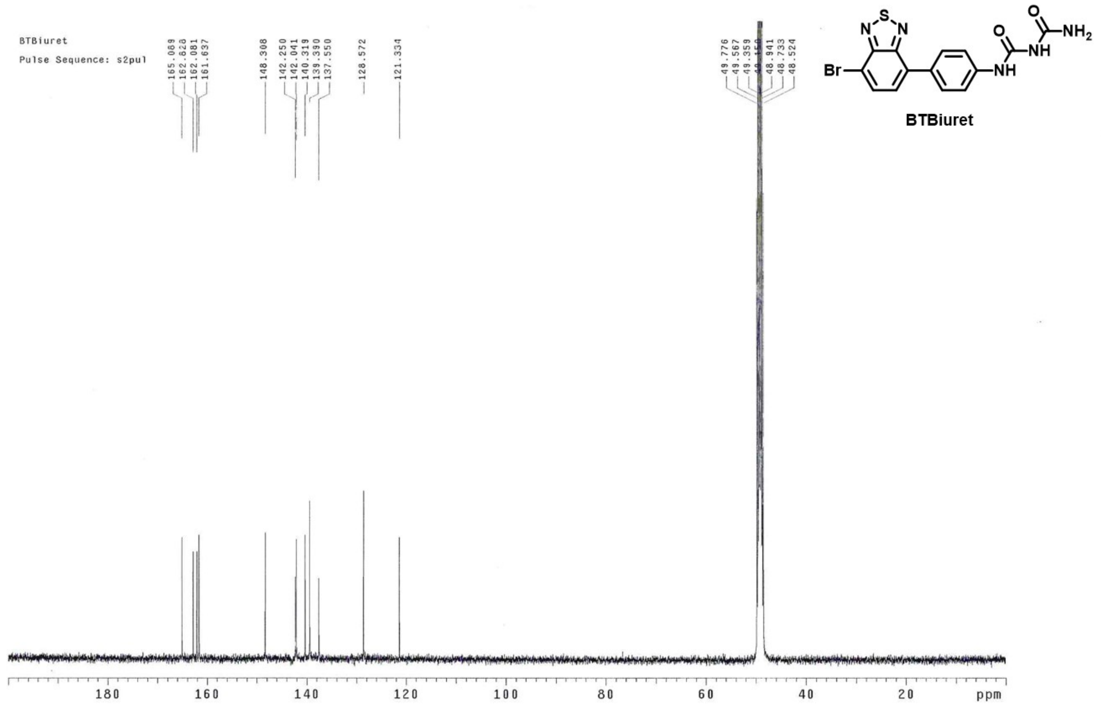

Figure S9. ^{1}H and ^{13}C spectra of compound **BTBiuret**.

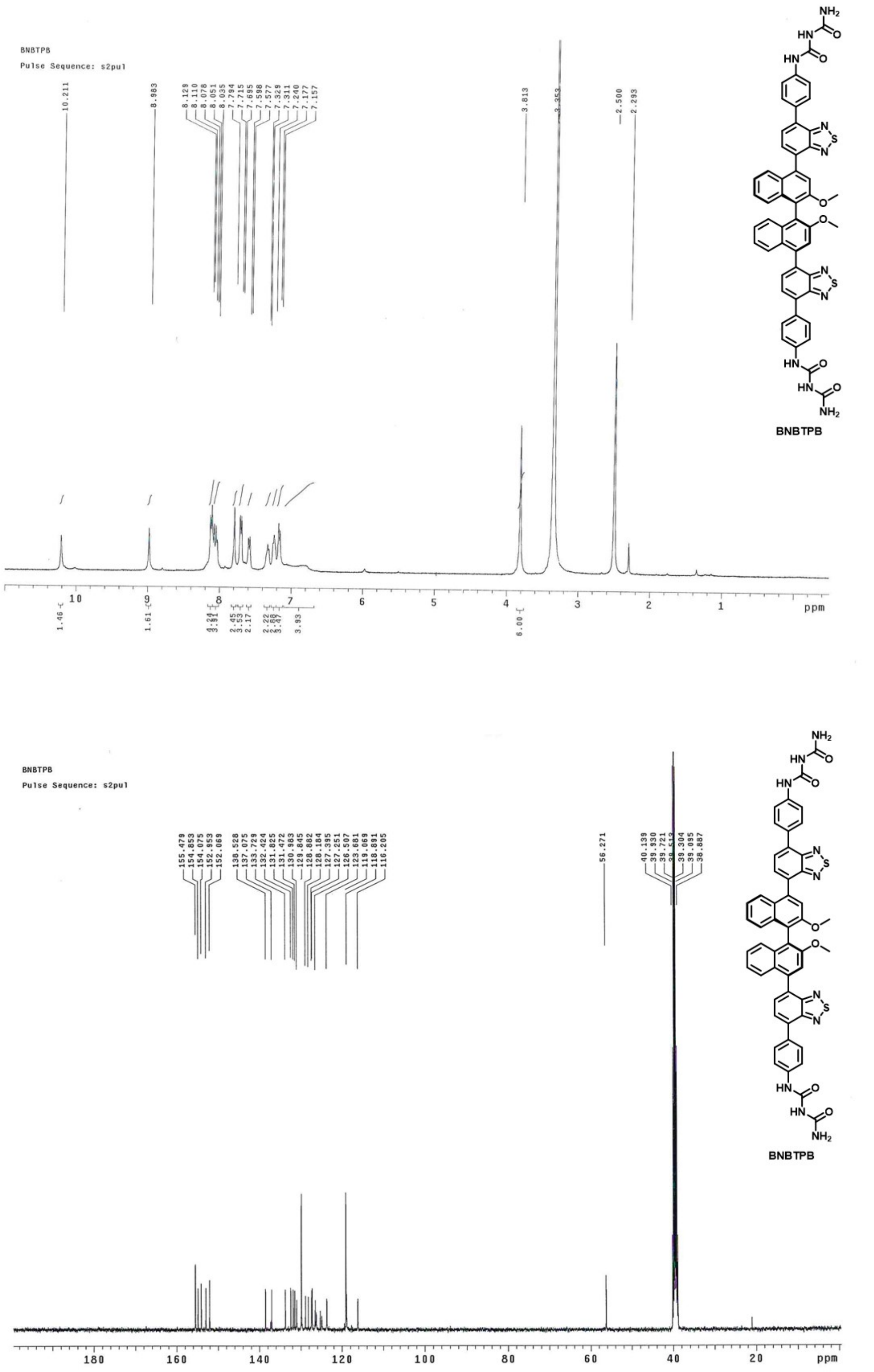

Figure S10. ^{1}H and ^{13}C spectra of compound **BNBTPB**.

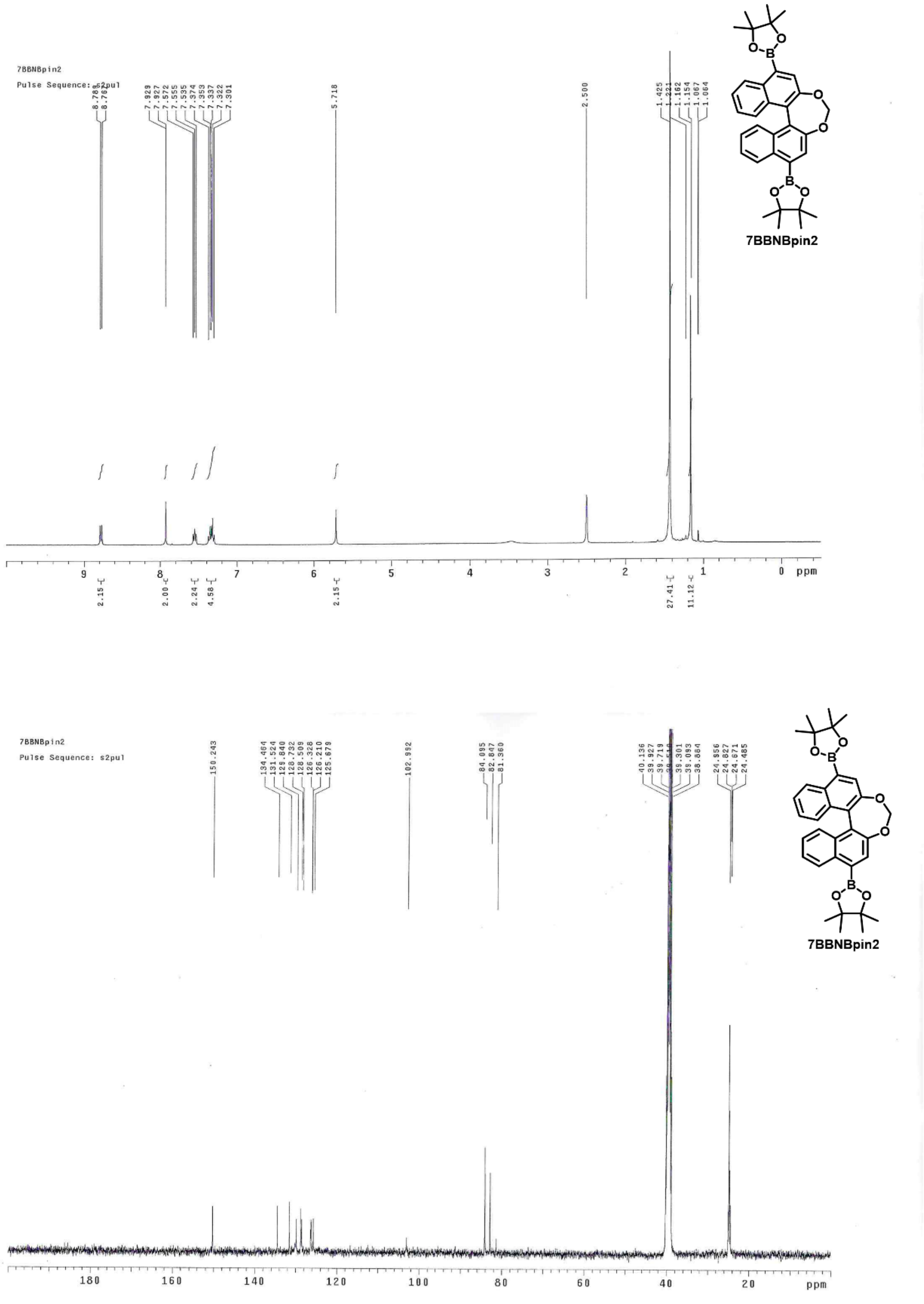

Figure S11. ^{1}H and ^{13}C spectra of compound **7BBNBpin2**.

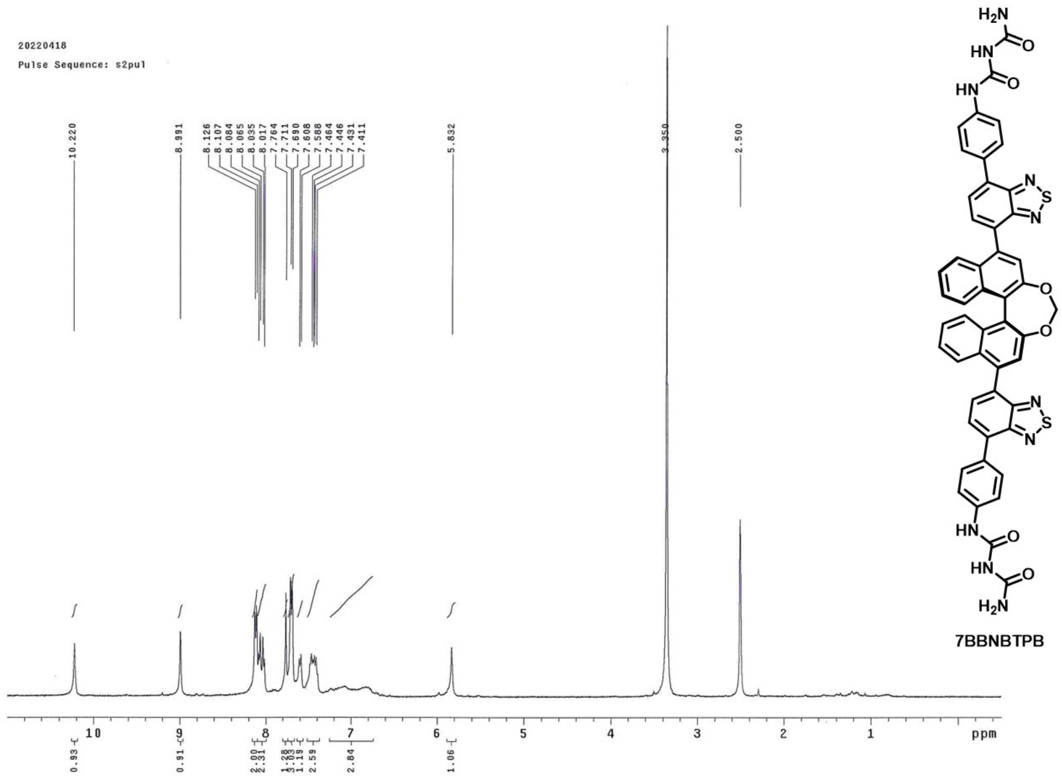

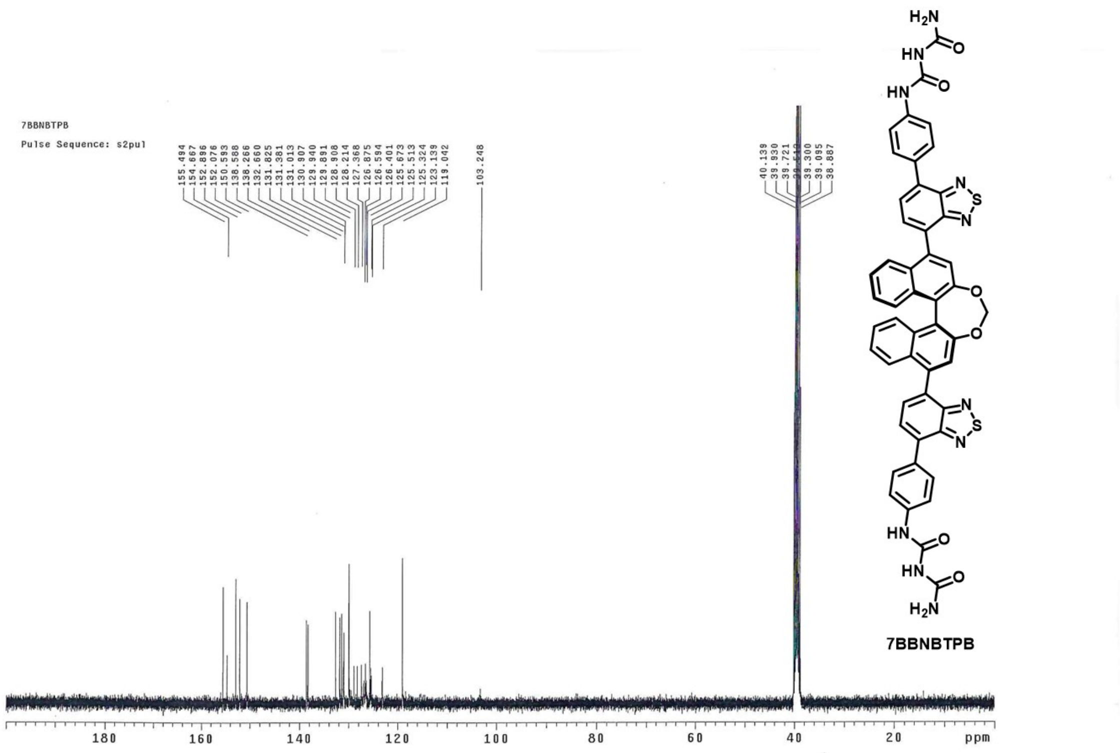

Figure S12. ^{1}H and ^{13}C spectra of compound **7BBNBTPB**.

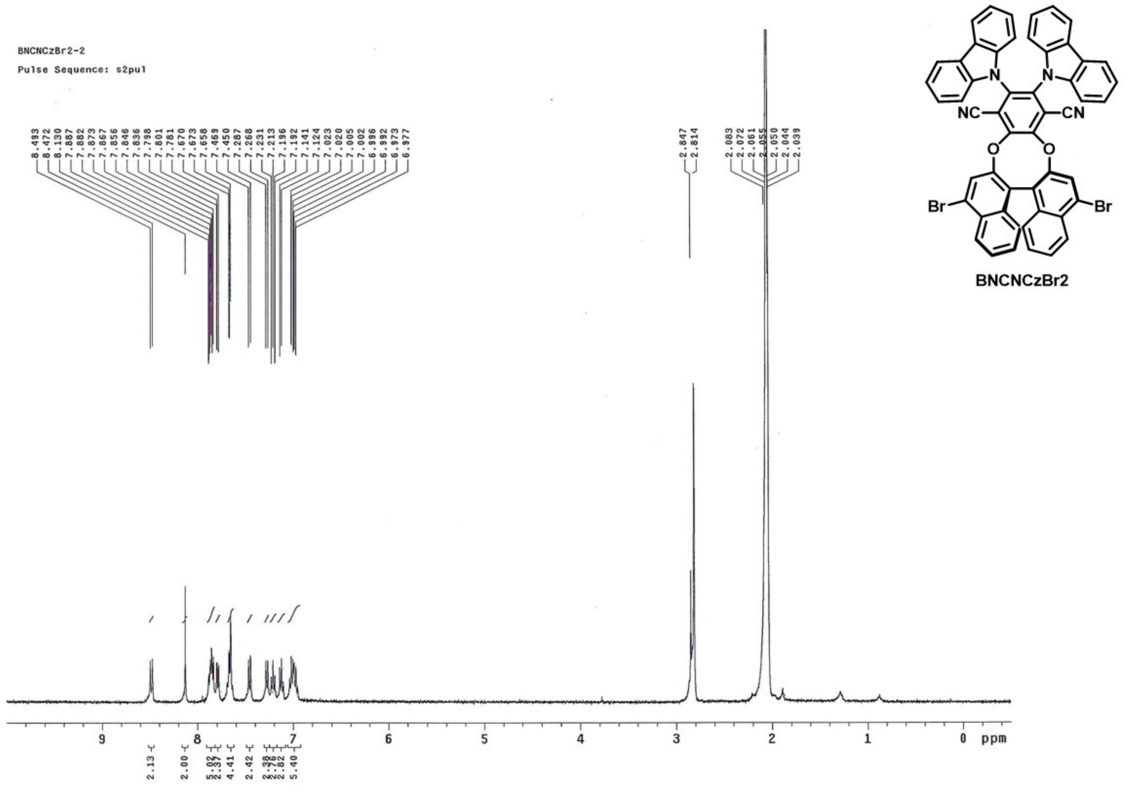

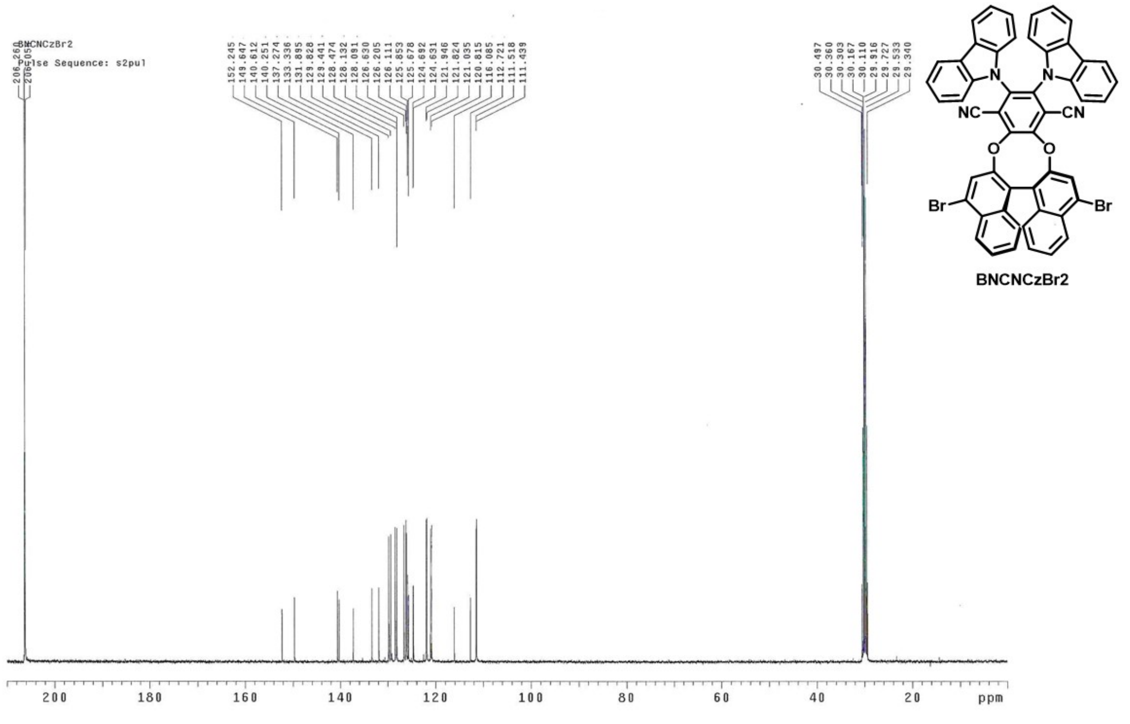

Figure S13. ^{1}H and ^{13}C spectra of compound **BNCNCzBr2**.

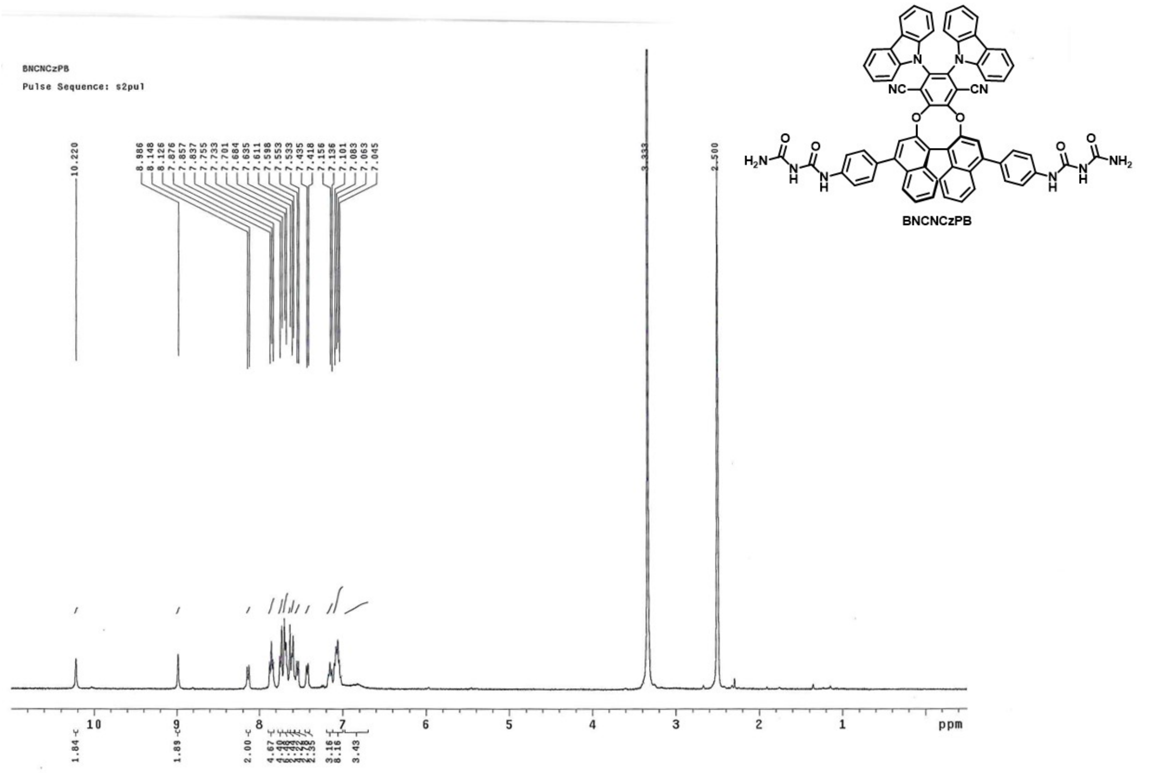

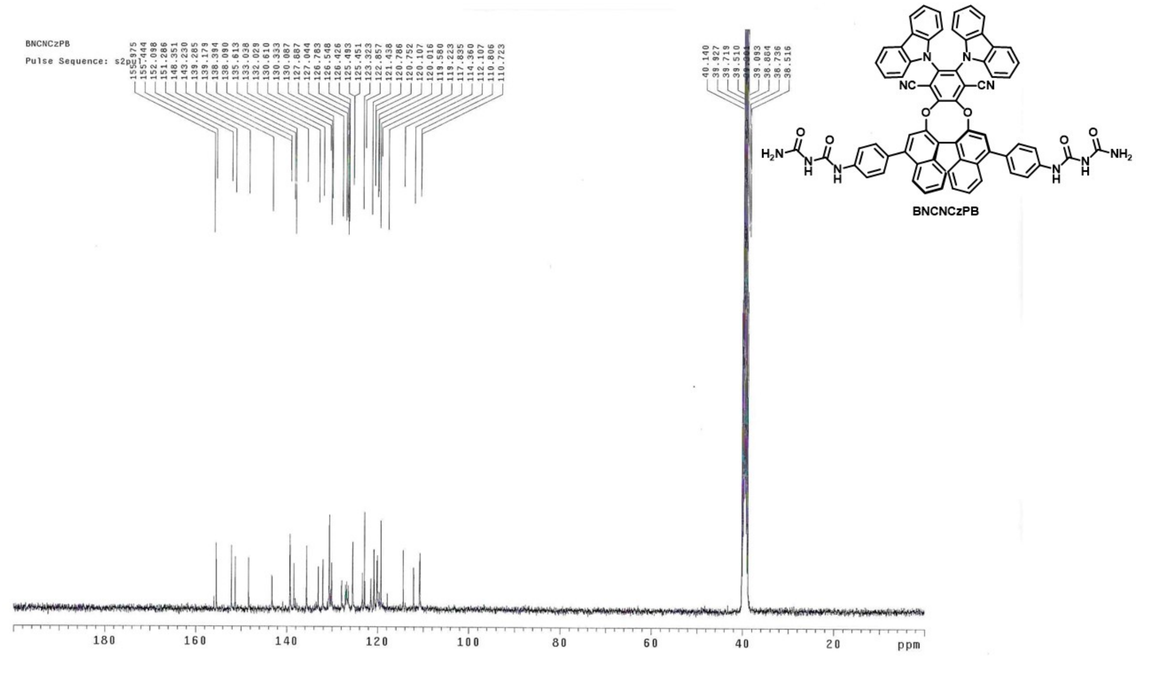

Figure S14. ^{1}H and ^{13}C spectra of compound **BNCNCzPB**.

4. References

1. MacLean, M.W.A.; Wood, T.K.; Wu, G.; Lemieux, R.P.; Crudden, C.M., Chiral Periodic Mesoporous Organosilicas: Probing Chiral Induction in the Solid State. *Chem. Mater.* **2014,** 26, 5852-5859, DOI: 10.1021/cm501826b.
